# Supplementary material for: Ground-state electron transfer in all-polymer donor:acceptor blends enables aqueous processing of water-insoluble conjugated polymers
Source: Nat Commun. 2023 Dec 20;14:8454. doi: 10.1038/s41467-023-44153-7 (PMC10730874; doi:10.1038/s41467-023-44153-7)
Supplement: Supplementary file 1 — Supplementary Information [file 41467_2023_44153_MOESM1_ESM.pdf]

Supplementary Information

Ground-state electron transfer in all-polymer donor:acceptor blends enables aqueous processing of water-insoluble conjugated polymers

Tiefeng Liu[1,2], Johanna Heimonen[1], Qilun Zhang[1], Chi-Yuan Yang[1,3], Jun-Da Huang[1,3], Han-Yan Wu[1], Marc-Antoine Stoeckel[1,3], Tom van der Pol[1], Yuxuan Li[4], Sang Young Jeong[5], Adam Marks[6], Xin-Yi Wang[7], Yuttapoom Puttisong[4], Asaminew Y. Shimolo[1], Xianjie Liu[1], Silan Zhang[1], Qifan Li[1], Matteo Massetti[1], Weimin M. Chen[4], Han Young Woo[5], Jian Pei[7], Iain McCulloch[6], Feng Gao[4], Mats Fahlman[1], Renee Kroon[1], Simone Fabiano[1,2,3,*]

[1]Laboratory of Organic Electronics, Department of Science and Technology, Linköping University, SE-601 74 Norrköping, Sweden.

[2]Wallenberg Initiative Materials Science for Sustainability, Department of Science and Technology, Linköping University, SE-601 74 Norrköping, Sweden.

[3]n-Ink AB, Bredgatan 33, SE-602 21 Norrköping, Sweden.

[4]Electronic and Photonic Materials, Department of Physics, Chemistry, and Biology, Linköping University, SE-581 83 Linköping, Sweden.

[5]Department of Chemistry, College of Science, Korea University, 136-713 Seoul, Republic of Korea.

[6]Department of Chemistry, University of Oxford, Oxford OX1 3TA, UK.

[7]Beijing National Laboratory for Molecular Sciences (BNLMS), Key Laboratory of Polymer Chemistry and Physics of Ministry of Education, Center of Soft Matter Science and Engineering, College of Chemistry and Molecular Engineering, Peking University, 100871 Beijing, China.

Correspondence should be addressed to: simone.fabiano@liu.se

Synthesis of PCAT-K

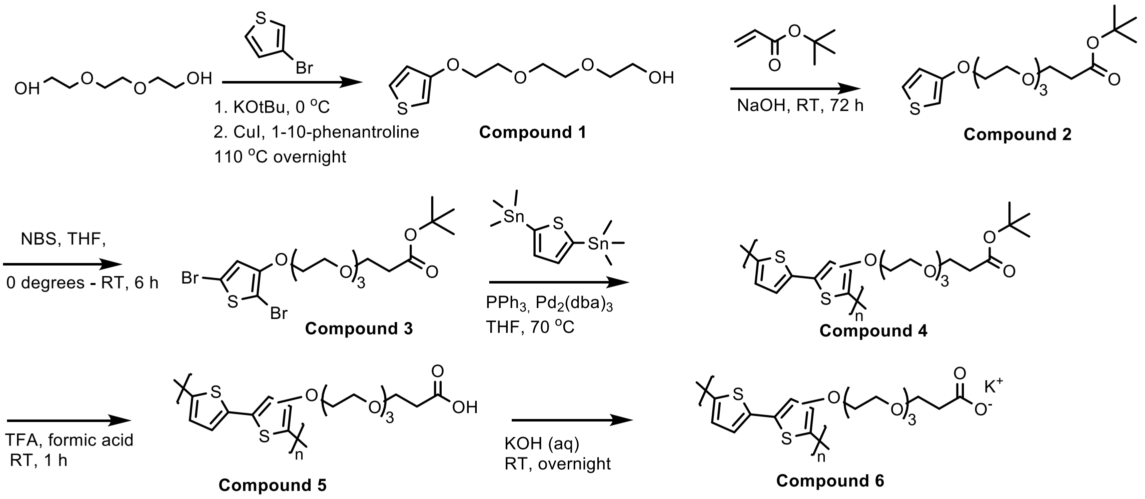

Compound 1

Ullman aryl ether synthesis

7.05 g (mmol, 7.5 eq) of triethylene glycol was added to a 25 ml round-bottom flask and lowered into a 0 °C ice bath under a nitrogen atmosphere. 1.03 g of potassium tert-butoxide was added in portions, and the mixture was stirred for 10 min. The vessel was transferred to an oil bath and stirred for 50 min at RT. The bath temperature was increased to 50 °C for 10 min. Then, 55 mg (0.61 mmol, 0.1 eq) of CuI, 220.4 mg of 1,10-phenantroline, and 0.57 ml (1 g, 6.1 mmol, 1 eq) of 3-bromothiophene were added, after which the mixture was stirred at 110 °C overnight. The reaction mixture was transferred to a 500 ml round-bottom flask and the vessel was washed with MeOH. 50 ml of silica gel was added with diethyl ether to form a slurry. The solvents were evaporated and the sample was dried in vacuo for 1.5 h. The product was purified on a silica gel column with 95:5 % Et2O:MeOH as the eluent. Fractions containing the product were combined, the solvent evaporated, and the product was stored in the fridge. The final yield was 0.88 g (62%). (1H NMR (500 MHz, CDCl3) δ 7.16 (dd, J = 5.3, 3.1 Hz, 1H), 6.78 (dd, J = 5.2, 1.5 Hz, 1H), 6.26 (dd, J = 3.2, 1.6 Hz, 1H), 4.12 (dd, J = 5.7, 3.8 Hz, 2H), 3.89 – 3.79 (m, 2H), 3.70 (dddd, J = 14.0, 8.3, 6.0, 4.3 Hz, 7H), 3.64 – 3.58 (m, 2H)), (13C NMR (126 MHz, cdcl3) δ 157.82, 125.02, 119.88, 97.91, 72.79, 71.09, 70.69, 70.01, 69.83, 62.07).

Compound 2

Oxa-Michael addition

653.4 mg (2.8 mmol, 1 eq) of compound 1 was combined with 451.5 mg (3.5 mmol, 1.3 eq) of tert-butyl acrylate and 8 mg of NaOH in a 10 ml round-bottom flask. The sample was shielded from light and stirred at RT for 72 h. The product was purified on a silica gel column and eluted with 25 % acetone in petroleum ether. The solvent was removed and the product was dried in vacuo overnight, the final yield was 0.95 g (94%). (1H NMR (500 MHz, cdcl3) δ 7.16 (dd, J = 5.2, 3.1 Hz, 1H), 6.77 (dd, J = 5.2, 1.6 Hz, 1H), 6.26 (dd, J = 3.1, 1.5 Hz, 1H), 4.13 – 4.09 (m, 2H), 3.87 – 3.81 (m, 2H), 3.75 – 3.57 (m, 10H), 2.50 (t, J = 6.6 Hz, 2H), 1.44 (s, 9H)), (13C NMR (126 MHz, cdcl3) δ 171.23, 157.95, 124.95, 119.93, 97.85, 80.83, 77.61, 77.36, 77.10, 71.14, 70.99, 70.90, 70.71, 70.03, 69.92, 67.24, 36.61, 28.43).

Compound 3

Bromination

In a typical procedure, 0.5072 g (1.41 mmol, 1 eq) of compound 2 was dissolved in 10 ml of tetrahydrofuran under a nitrogen atmosphere and sparged for 30 min. The solution was allowed to cool to 0 °C for 15 min before 0.50 g (2.82 mmol, 2 eq) of N-bromo succinimide was added portion wise. The mixture was reacted for 1.5 h at 0 °C, an additional portion of N-bromo succinimide was added after 5 hours to drive the reaction to completion, after which the reaction was allowed to warm to RT and react overnight at RT. After 5 h, an. The reaction was neutralized by addition of 5 ml 1 M sodium bicarbonate and stirred for 15 min. The solution was transferred to an extraction flask along with 140 ml of diethyl ether. The organic phase was extracted with 1x50 ml of 1 M sodium bicarbonate and 3x50 ml of brine. The water phase was extracted with 2x50 ml of diethyl ether. The organic phases were combined and dried over sodium sulphate and magnesium sulphate. The solvent was removed by evaporation and the product was dried in vacuo overnight. The product was purified on a silica gel column and eluted with 20 % acetone in petroleum ether. The eluate was collected in fractions and monitored by TLC. The final yield was 0.35 g (45%) of pure product which was collected as a yellow oil. ($_{1}$H NMR (500 MHz, cdcl3) δ 6.82 (s, 1H), 4.17 – 4.13 (m, 2H), 3.81 – 3.76 (m, 2H), 3.74 – 3.68 (m, 4H), 3.67 – 3.59 (m, 6H), 2.50 (t, J = 6.6 Hz, 2H), 1.44 (s, 9H)), ($_{13}$C NMR (126 MHz, cdcl3) δ 171.25, 154.09, 121.85, 109.93, 72.39, 71.34, 71.02, 70.93, 70.75, 70.17, 67.26, 36.63, 28.45).

Compound 4

Stille Coupling

A dried 50 mL round-bottom flask was loaded with compound 4 (2 g, 3.86 mmol) and 2,5-bis(trimethylstannyl)thiophene (1.58 g, 3.86 mmol, 1.0 eq) and dissolved in dry, sparged (30 min with N_2) tetrahydrofuran (250 mL). Then, tris(dibenzylideneacetone)dipalladium(0) (77.2 mg, 2 mol-%), triphenylphosphine (83 mg, 8 mol-%) were added under nitrogen atmosphere. After dissolving, the solution was heated to 70 °C under stirring, during which the color progressed to deep red and the viscosity increased. After 48 hours reaction time, the solution was cooled to room temperature and poured into hexanes under vigorous stirring which yielded blue fibers. After filtration, the polymer was redissolved in chlofororm and vigorously stirred with a 5% m/v solution of sodium diethyldithiocarbamate trihydrate in deionized water at reflux under N_2. After liquid-liquid extraction, the chloroform solution was reduced in volume, precipitated in heptane and transferred to a Soxhlet extraction thimble. The polymer was Soxhlet extracted with heptane until each washing yielded a clear extract. After collecton with isopropanol and drying the polymer solution *in vacuo*, the polymer was precipitated in isopropanol, dried under vacuum to yield 1.34 grams (82%) of the target material as a blue sticky material. (1H-NMR (500 MHz, cdcl3) δ 7.25-6.83 (m, 3H), 4.39-4.22 (m, 2H), 3.97-3.40 (m, 12H), 2.55-2.35 (m, 2H), 1.49-1.28 (2, 9H)). 1H-NMR spectra was presented in Supplementary Figure 1.

Compounds 5 and 6

Acid catalyzed deprotection and formation of the polyanion

1 g of compound 4 was dissolved in a sparged mixture of 15 ml of formic acid and 4 ml of trifluoroacetic acid before being stirred overnight at room temperature under nitrogen atmosphere. The polymer was precipitated in water and centrifuged at 3500 rpm for 10 min. The supernatant was discarded, and the pellet was washed with additional water 3 times before the product was collected. Compound 5 is an insoluble dark purple solid, the yield of compound 5 was not determined. Compound 5 was added to a round-bottom flask along with 150 ml of water and 1 g of potassium hydroxide forming a purple viscous solution which was stirred at RT overnight. The polymer was precipitated in acetone, resulting in dark purple particles that were collected via centrifugation. The particles were successively washed with acetone:water mixture (90:10) to remove excess potassium hydroxide and collected. This process yielded 0,993 g (quantitative) of a dark purple solid. ^{1}H-NMR indicated that the deprotection was successful, but could not supply in-depth structural characterization due to severe line broadening. 1H-NMR spectra was presented in Supplementary Figure 2.

Supplementary Note 1. Relative elemental concentrations of carbon, hydrogen, sulphur, oxygen and potassium were analyzed for PCAT-K, and were calculated from the chemical structure of the repeating unit. The difference between the calculated and found content can be explained by the hygroscopic nature of the PCAT-K, where ~20 mass % ($C_{17}H_{19}KO_6S_2 \cdot 5H_2O$) is represented by residual water despite a 24 hour drying step at 40 °C under vacuum (1 mbar).

**$C_{17}H_{19}KO_6S_2$**	**C [%]**	**H [%]**	**O [%]**	**S [%]**	**K [%]**
calculated	48.32	4.53	22.72	15.17	9.25
found	37.95	5.59	35.87	10.98	8.37
**$C_{17}H_{19}KO_6S_2 \cdot 5H_2O$**					
calculated	39.83	5.7	34.33	12.51	7.63

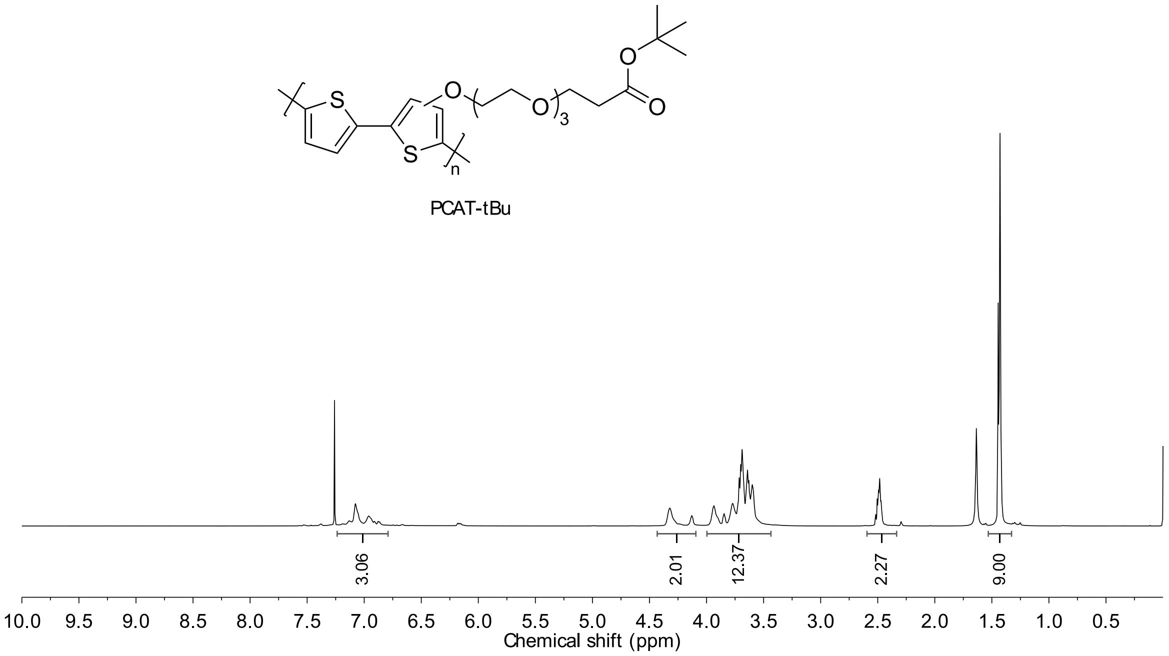

Supplementary Figure 1. The ^{1}H-NMR spectrum of compound 4 (PCAT-tBu).

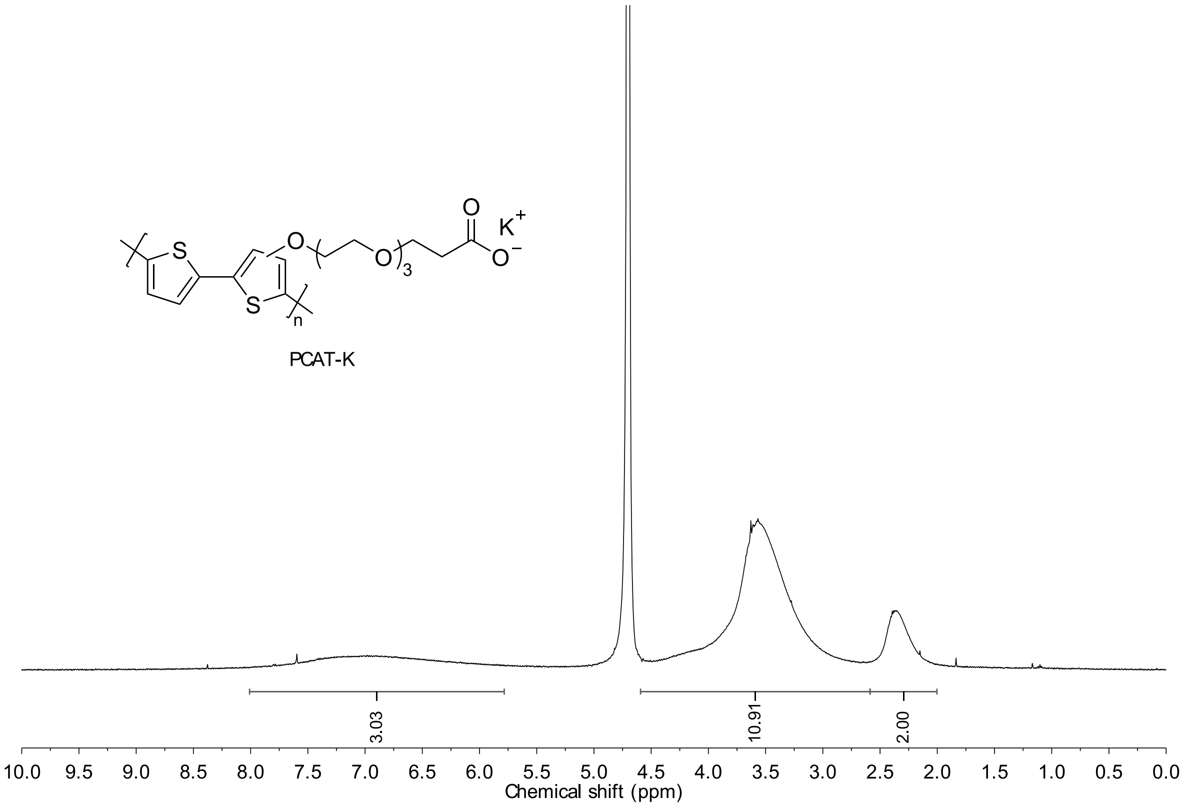

Supplementary Figure 2. The ^{1}H-NMR spectrum of PCAT-K.

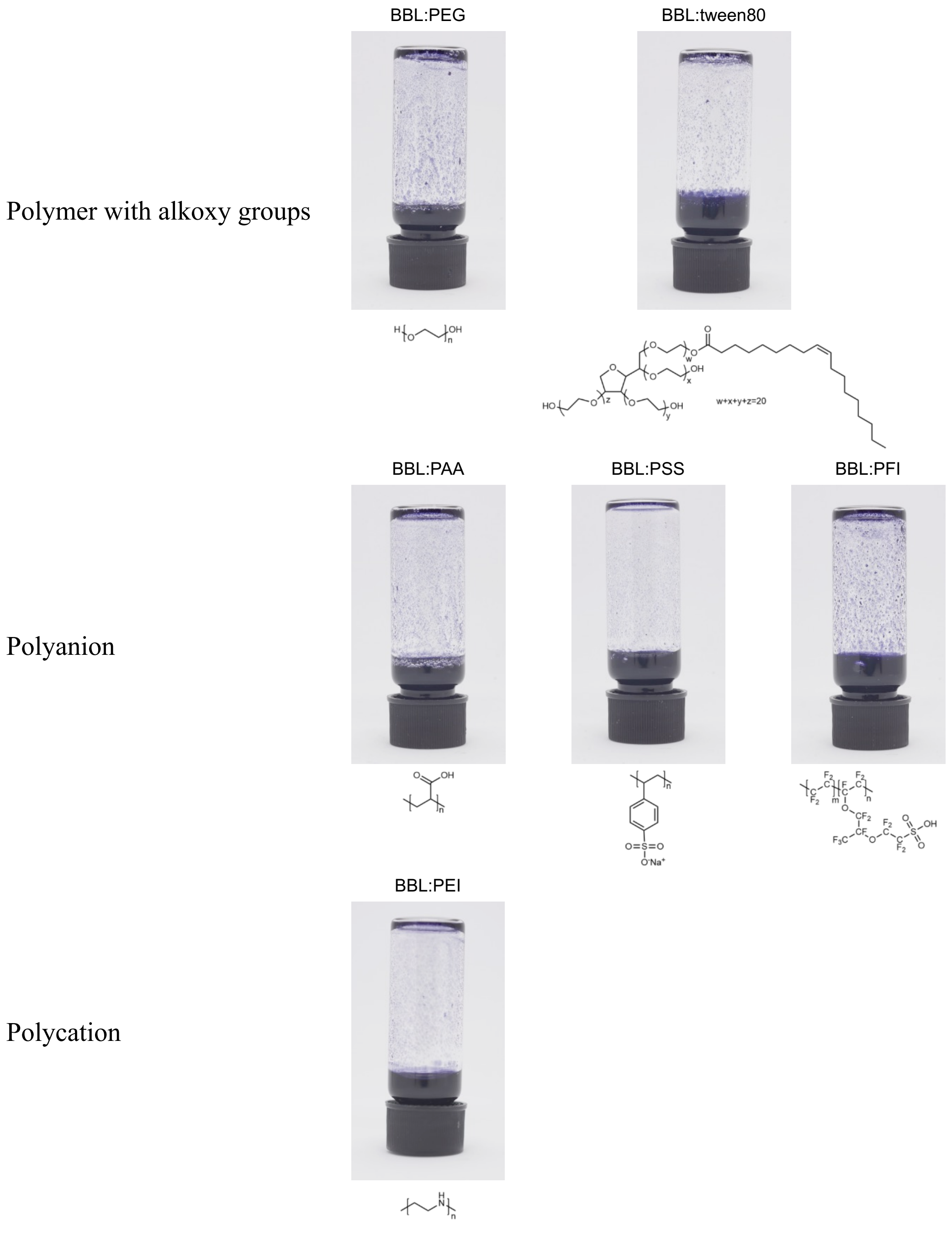

Supplementary Figure 3. Photographs of BBL dispersion in water in the presence of common anionic, cationic, and non-ionic surfactants: Poly(ethylene glycol) (PEG, M_n = 35 kDa)[1]. Polyethylene glycol sorbitan monooleate (TWEEN 80, M_w = 1.3 kDa)[2]. Poly(acrylic acid) (PAA, M_w = 100 kDa)[3]. Poly(sodium-4-styrene sulfonate) (PSS, M_w = 75 kDa)[4]. Perfluorinated sulfonic acid ionomers (PFI, M_w ~ 100 kDa)[5]. Linear poly(ethyleneimine) (PEI, M_n = 2.5 kDa)[6]. For all surfactants, the BBL:surfactant ratio is 1:1, and the total concentration is 2 mg/ml in water. The surfactant chemical structures are presented below the pictures.

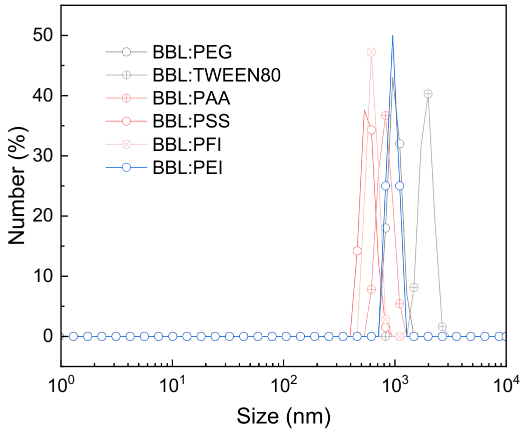

Supplementary Figure 4. Particle size distribution of the different BBL:surfactant dispersions.

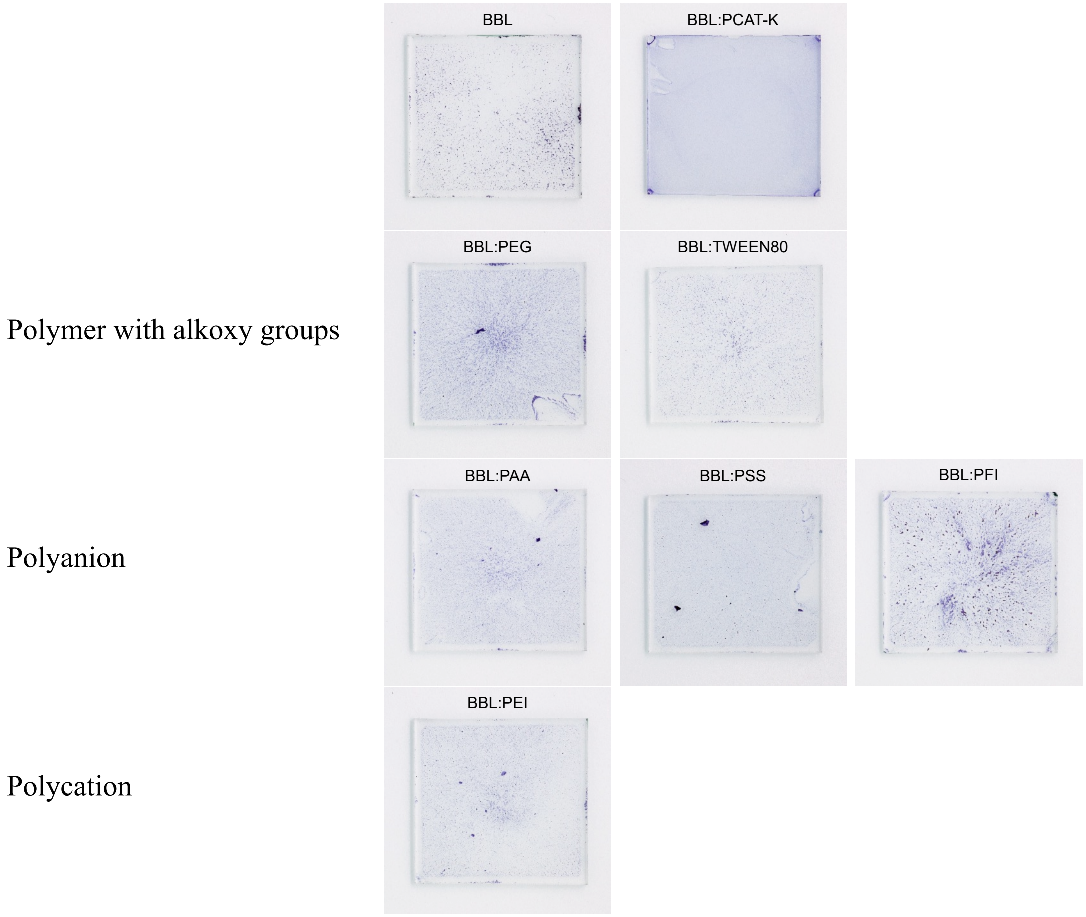

Supplementary Figure 5. Photographs of the spin-cast BBL:surfactant films (solution reported in Supplementary Figure 3). The dispersions were spin-cast on plasma-treated glass slides at 1,000 r.p.m., followed by annealing at 150 °C for 10 min in ambient. The glass size is about 2.5 × 2.5 cm^2.

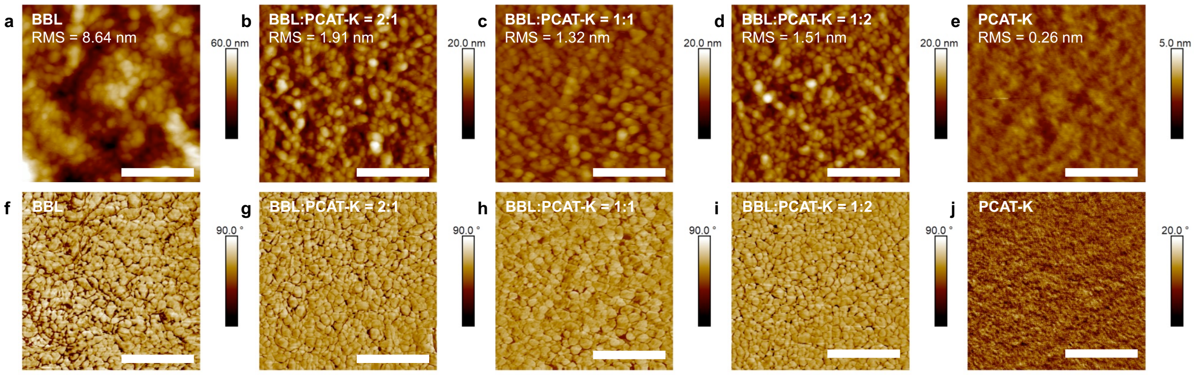

Supplementary Figure 6. Atomic force microscopy images of thin films of BBL, PCAT-K, and BBL:PCAT-K with different ratios on glass substrates: (**a**-**e**) height images and (**f**-**j**) phase images. The scale bar is 400 nm.

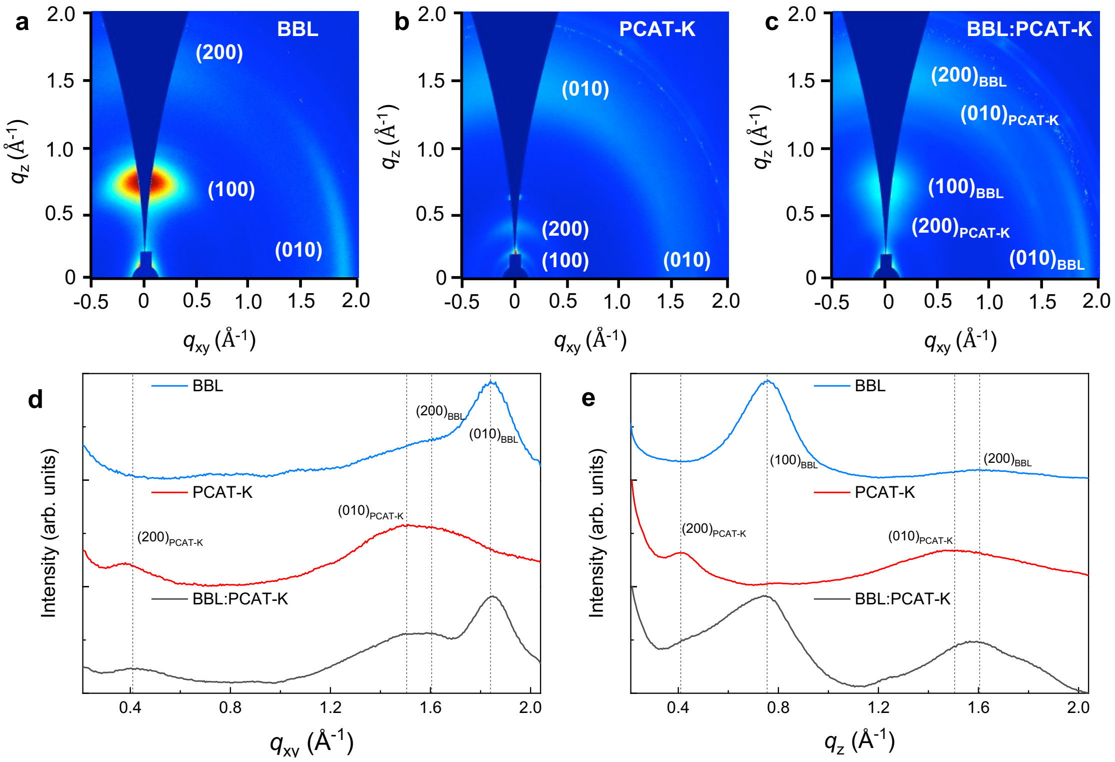

Supplementary Figure 7. 2D GIWAXS patterns of (**a**) BBL, (**b**) PCAT-K, and (**c**) BBL:PCAT-K. In-plane (**d**) and out-of-plane (**e**) GIWAXS line cuts of BBL, PCAT-K, and BBL:PCAT-K films. The mass ratio of BBL:PCAT-K is 1:1.

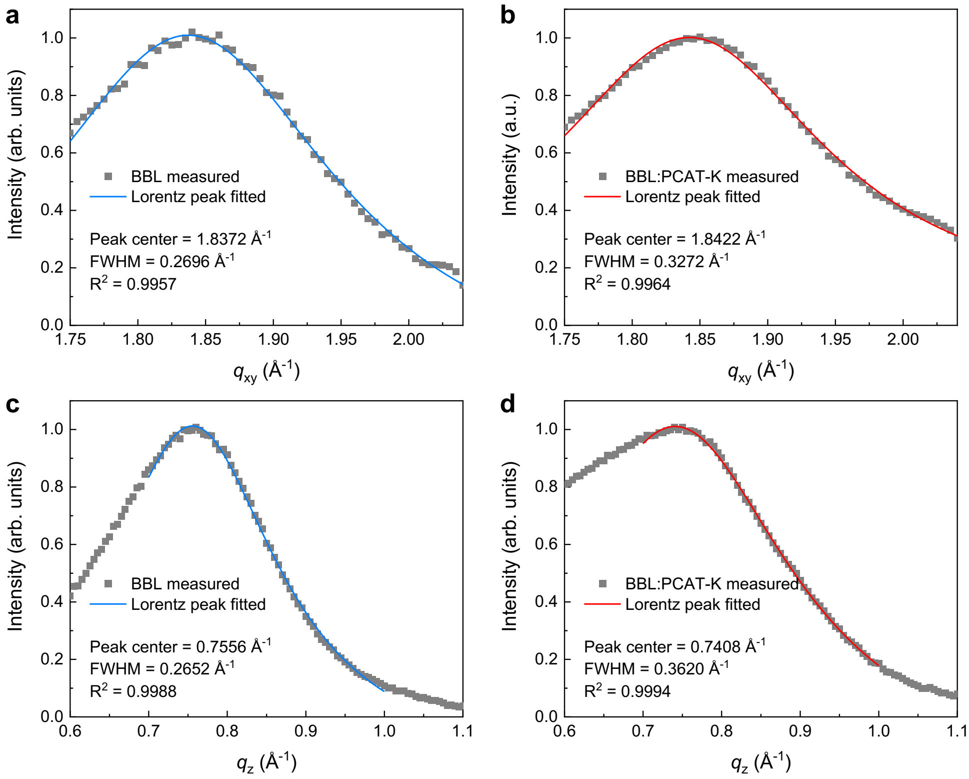

Supplementary Figure 8. π-π stacking (010) diffraction analysis of (**a**) BBL and (**b**) BBL:PCAT-K. Lamellar (100) diffraction analysis of (**c**) BBL and (**d**) BBL:PCAT-K. Pristine BBL shows a predominant edge-on orientation with respect to the substrate, with a strong lamellar (100) peak at $q_z = 0.756$ Å^{-1} (*d*-spacing = 8.32 Å) and a strong π-π stacking (010) peak at $q_{xy} = 1.837$ Å^{-1} (*d*-spacing = 3.42 Å) while the PCAT-K shows a mixed edge-on and face-on orientation. On the contrary, the BBL:PCAT-K blend film shows a weak diffraction pattern. From the linecuts, the diffraction peaks relative to BBL and PCAT-K can be observed in the blend film with the weak intensity and broad width, indicative of a good intermixing between the two polymers and a poor aggregation. Particularly, the coherence lengths of BBL π-π stacking and lamellar packing in the blend film decrease from 20.98 Å to 17.28 Å and 21.32 Å to 15.62 Å, respectively.

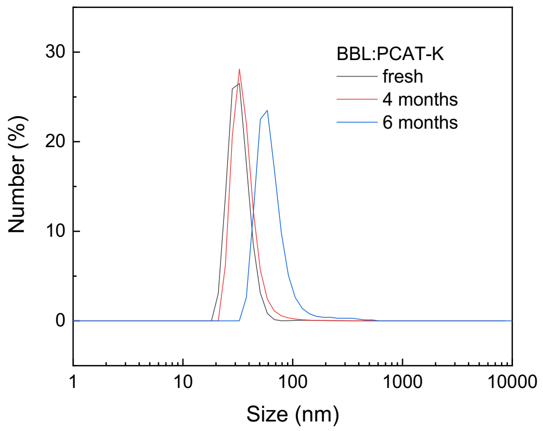

Supplementary Figure 9. Particle size distribution of a water-based BBL:PCAT-K dispersion stored in ambient.

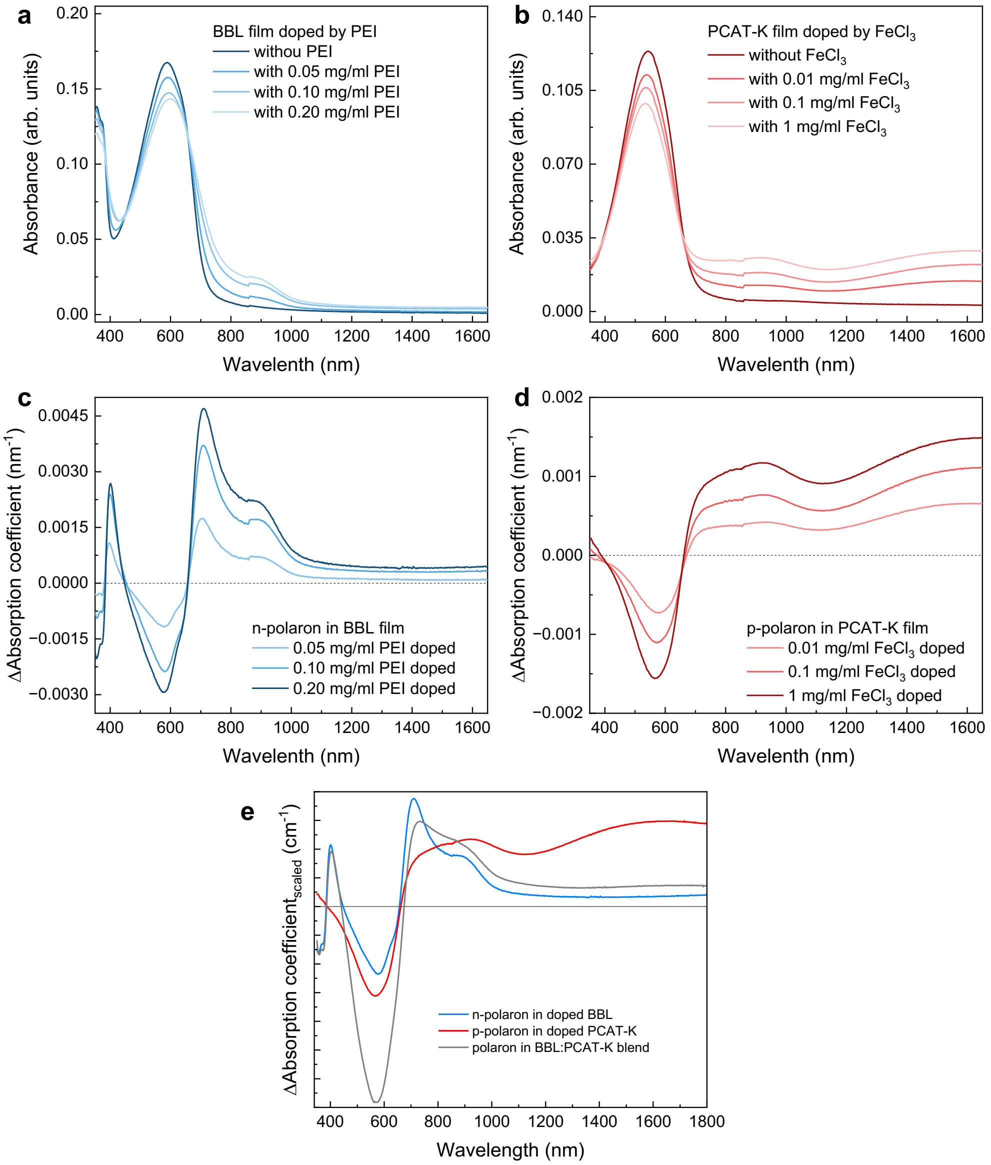

Supplementary Figure 10. UV-Vis-NIR absorption spectra of (**a**) PEI-doped BBL and (**b**) $FeCl_3$-doped PCAT-K films. The differential absorption coefficient spectra of (**c**) PEI-doped BBL and (**d**) $FeCl_3$-doped PCAT-K films. (**e**) Comparison of differential absorption coefficient of n-polaron in doped BBL, p-polaron in doped PCAT-K, and polaron in BBL:PCAT-K blend. BBL exhibits characteristic polaronic peaks at low wavelengths (700-1000 nm), while PCAT-K shows several broad features at both lower (800-1100 nm) and higher wavelengths (>1200 nm). The BBL's polaron absorption shows a distinct peak at 710 nm along with a vibrational progression, which is also evident in the BBL:PCAT-K spectra. However, the peak ratio differs significantly in the BBL:PCAT-K blend. The absorption peak at higher wavelengths (*i.e.*, 880 nm) is more pronounced compared to PEI-doped BBL, reminiscent of the polaron spectrum of PCAT-K. Therefore, we concluded that the differential spectra of BBL:PCAT-K are dominated by the BBL polaron but also include contributions from the PCAT-K polaron.

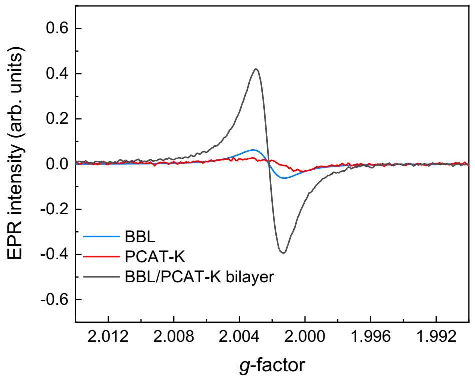

Supplementary Figure 11. EPR signals of BBL, PCAT-K, and BBL/PCAT-K bilayer films.

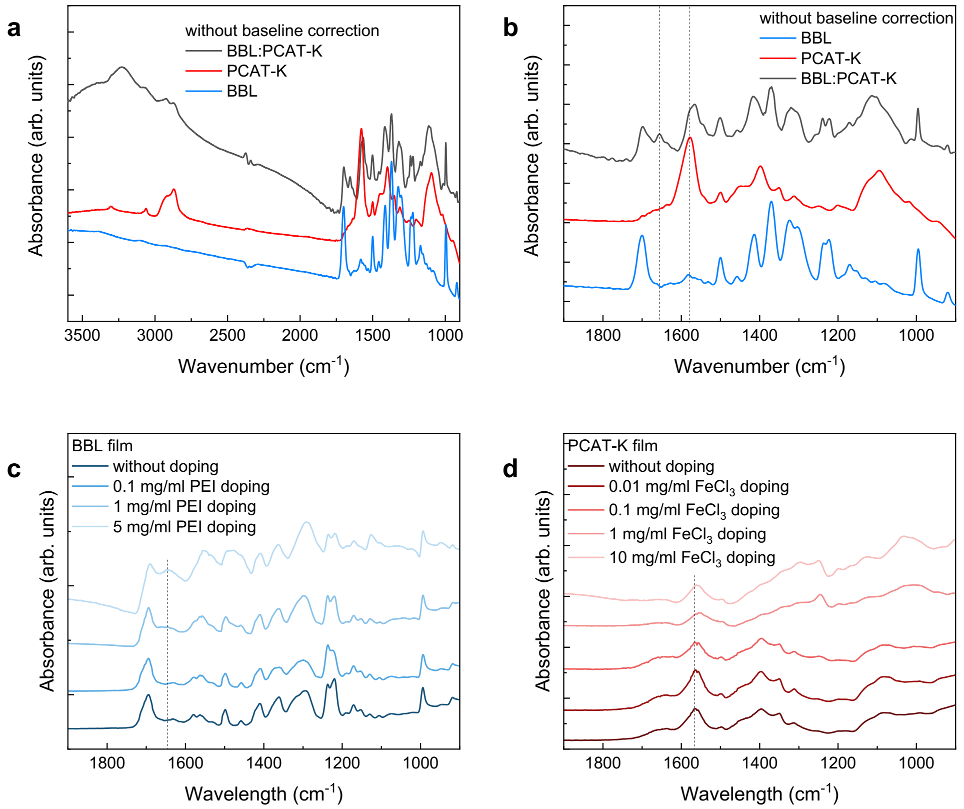

Supplementary Figure 12. (**a**,**b**) FTIR absorption spectra of BBL, PCAT-K, and BBL:PCAT-K films. FTIR absorption spectra of (**c**) PEI-doped BBL and (**d**) $FeCl_3$-doped PCAT-K films. A broad absorption band in the range 2000-3500 cm^{-1} is visible for BBL:PCAT-K films and attributed to polarons[7]. A new peak at 1655 cm^{-1}, assigned to antisymmetric C=O stretching, appears in the FTIR spectrum of BBL:PCAT-K. This is the fingerprint of negative polarons in BBL[8]. The peak at 1578 cm^{-1}, assigned to the antisymmetric $C_\alpha = C_\beta$ bonds stretching of PCAT, shifts to lower wavenumbers, indicating doping of PCAT-K[9]. These shift are comparable to the peak shifts observed for the individually (chemically) doped BBL and PCAT-K, indicating that both BBL and PCAT-K are doped in the blend films.

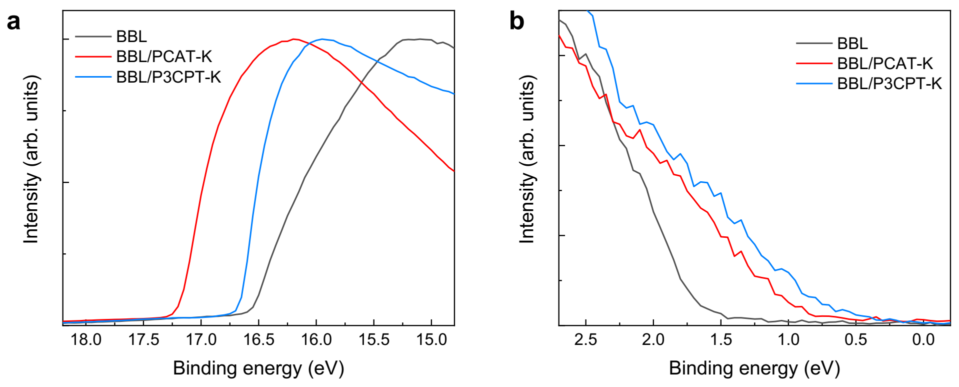

Supplementary Figure 13. UPS spectra of BBL (black), BBL/PCAT-K (red), and BBL/P3CPT-K (blue) films. (**a**) Secondary electron cut-off and (**b**) frontier valence regions. The starting substrate is gold for all cases. The work function (WF) can be determined from the secondary electron cut-off and is 4.63 eV for the BBL films. When PCAT-K and P3CPT-K are deposited onto BBL to form bilayer structures, the chemical potential is equilibrated across the bilayers. If electron transfer occurs in this process, a shift of the secondary electron cut-off is generated in the UPS spectra that signifies a corresponding shift of the vacuum level at the interface. The PCAT-K film shows a large shift in the secondary electron cut-off when deposited on top of the BBL film, which is in agreement with substantial electron transfer from PCAT-K to BBL. Only a near-negligible shift in the secondary electron cut-off is seen for P3CPT-K on BBL, suggesting the absence of significant GSET.

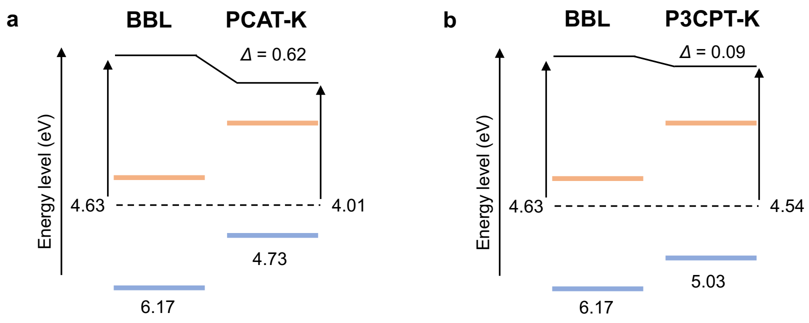

Supplementary Figure 14. The resulting down-shift (Δ, in eV) of the vacuum level for (**a**) BBL/PCAT-K and (**b**) BBL/P3CPT-K heterojunctions is reported in the diagram.

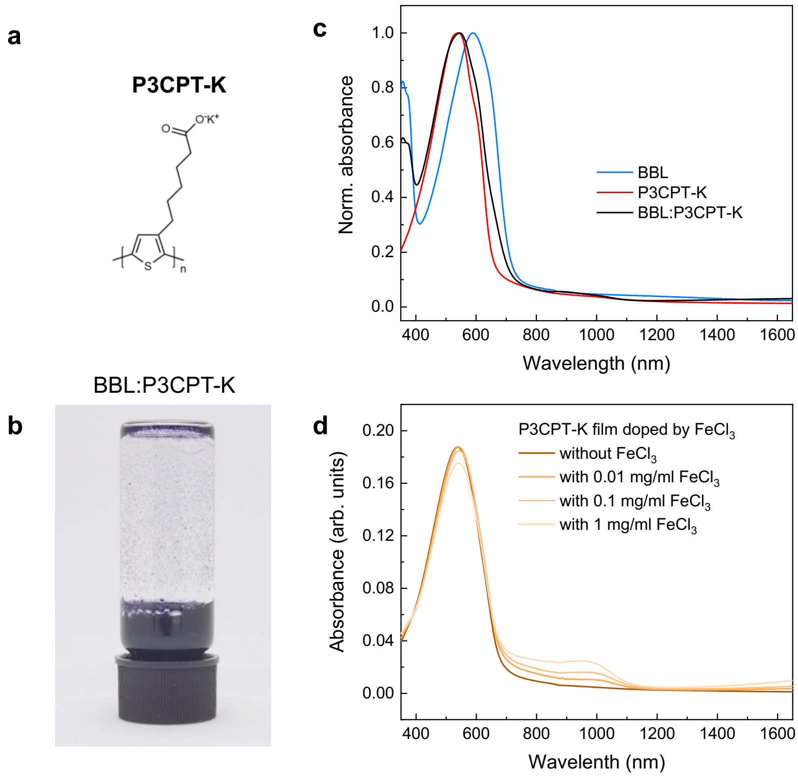

Supplementary Figure 15. (**a**) Chemical structure of P3CPT-K. (**b**) Photograph of a 1:1 BBL:P3CPT-K dispersion. (**c**) Normalized absorption spectra of BBL, P3CPT-K, and BBL:P3CPT-K films. (**d**) Absorption spectra of $FeCl_3$-doped P3CPT-K films.

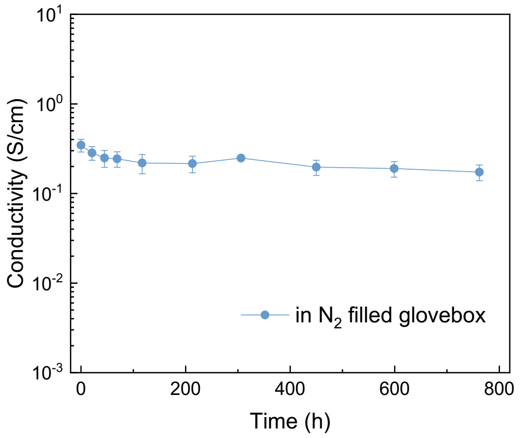

Supplementary Figure 16. Evolution of the electrical conductivity of BBL:PCAT-K films stored in an N_2-filled glovebox. Error bars indicate the SD of ten experimental replicates.

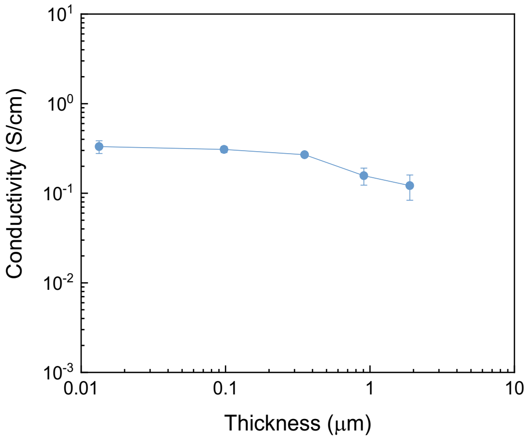

Supplementary Figure 17. Thickness-dependence of the conductivity of BBL:PCAT-K films. Thin films (< 100 nm) were deposited by spin-coating while thick films (> 100 nm) were drop-casted. Error bars indicate the SD of ten experimental replicates.

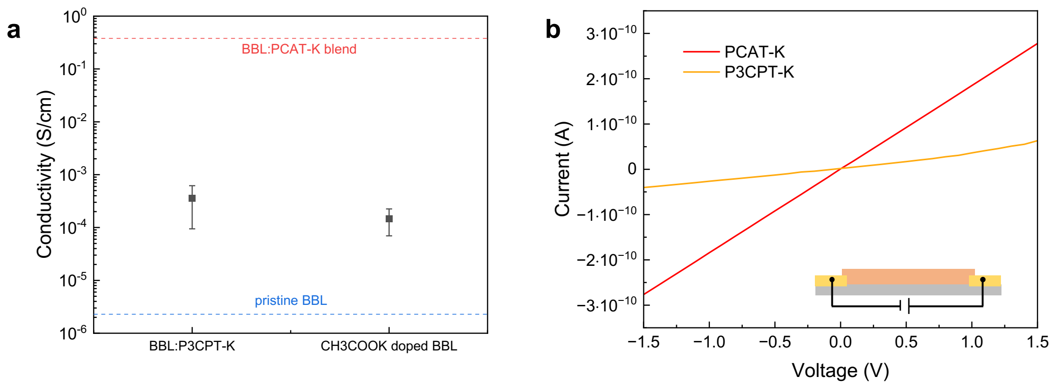

Supplementary Figure 18. (**a**) Conductivity of BBL:P3CPT-K and potassium acetate-doped BBL. (**b**) The current-voltage curve of pure PCAT-K and P3CPT-K films. The film thickness is about 40 nm, and the channel size (width to length) is 30 to 1000 μm.

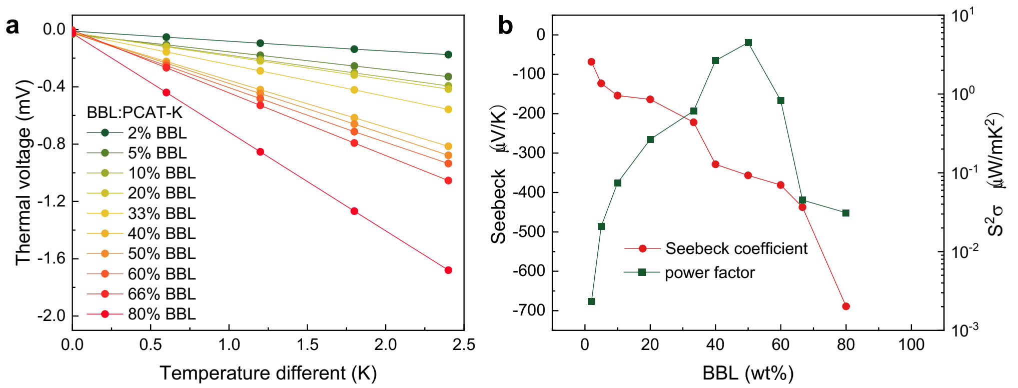

Supplementary Figure 19. (**a**) Thermovoltage of BBL:PCAT-K films at different BBL content. (**b**) Seebeck coefficient and power factor of BBL:PCAT-K films at different BBL content.

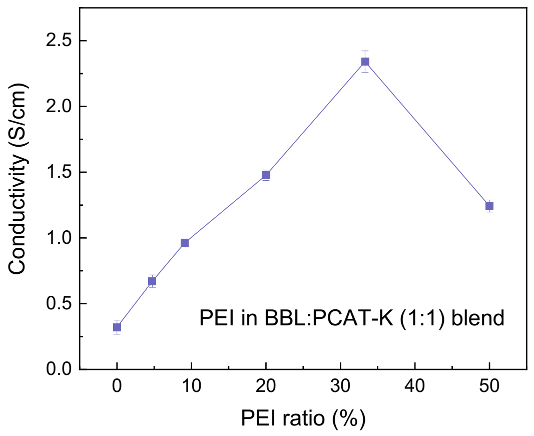

Supplementary Figure 20. Conductivity of BBL:PCAT-K films at different PEI content. Error bars indicate the SD of ten experimental replicates.

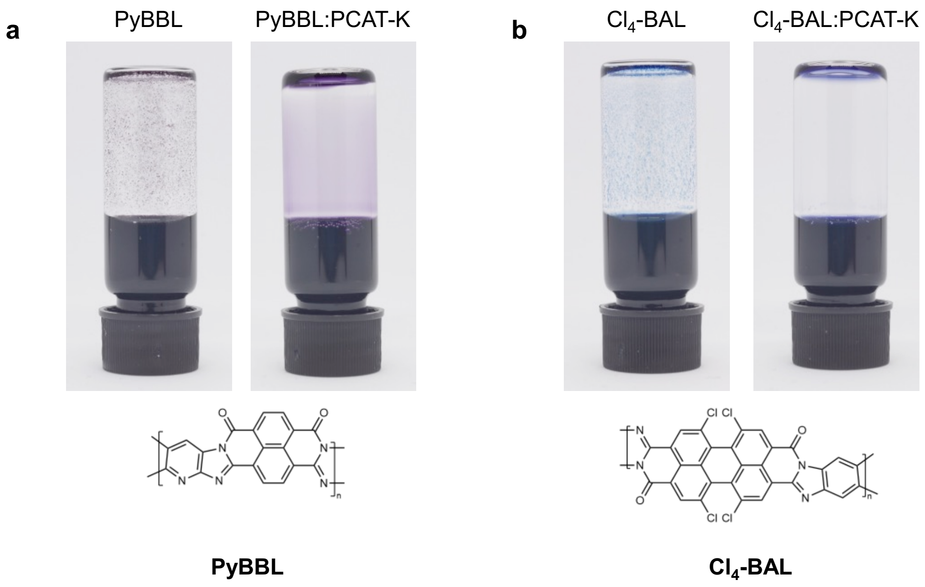

Supplementary Figure 21. (**a**) Photographs of PyBBL (1 mg/ml) and PyBBL:PCAT-K 1:1.5 (2.5 mg/ml) aqueous solutions. (**b**) Photographs of Cl_4-BAL (1 mg/ml) and Cl_4-BAL:PCAT-K 1:1 (2 mg/ml) aqueous solutions. The PyBBL and Cl_4-BAL water dispersions were prepared using the same solvent-exchange method used for BBL.

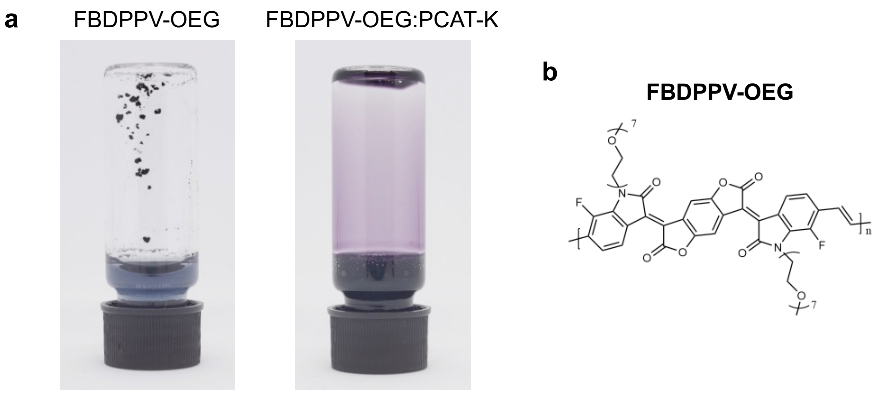

Supplementary Figure 22. (**a**) Photographs of FBDPPV-OEG (10 mg/ml) and FBDPPV-OEG:PCAT-K 1:1 (20 mg/ml) aqueous solutions and (**b**) chemical structure of FBDPPV-OEG.

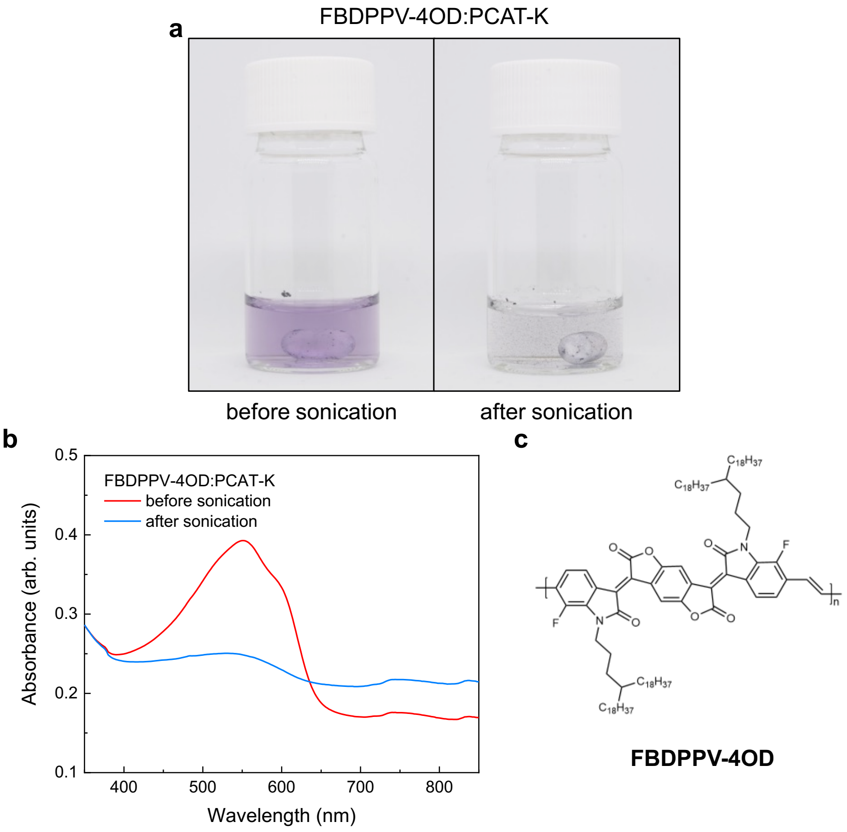

Supplementary Figure 23. (**a**) Photographs of FBDPPV-4OD:PCAT-K 1:1 (0.1 mg/ml) solution before and after 5 min of sonication. (**b**) Absorbance spectrum of their corresponding solution, showing the disappearance of PCAT-K from solution after sonication. (**c**) Chemical structure of FBDPPV-4OD.

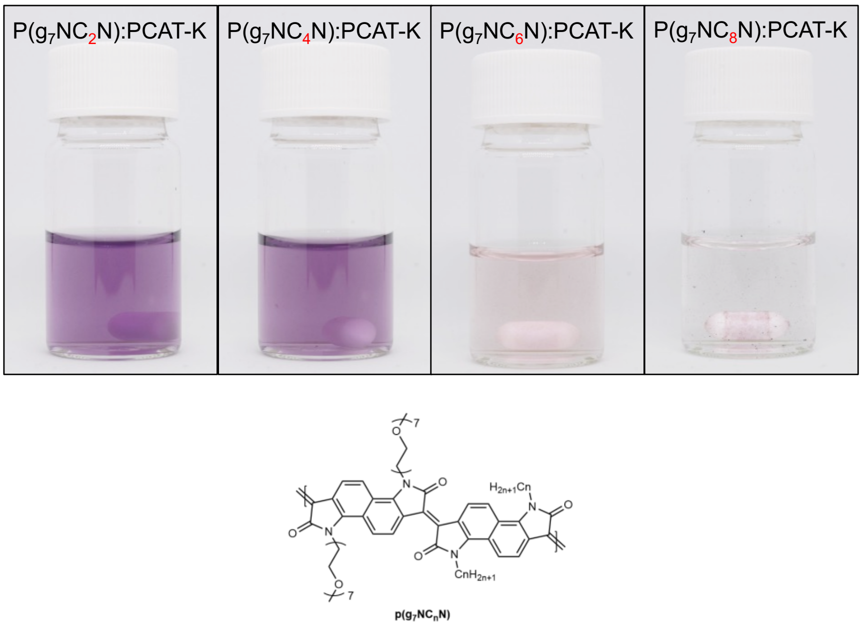

Supplementary Figure 24. Photographs of P(g_7NC$_n$N):PCAT-K (1:1) aqueous solutions. P(g_7NC$_n$N) has polar side chains and alkyl side chains with gradually increasing lengths (from C_2 to C_8). P(g_7NC$_n$N) with short alkyl side chains, like P(g_7NC$_2$N) and P(g_7NC$_4$N), can dissolve in water in the presence of PCAT-K, while P(g_7NC$_n$N) with long alkyl side chain, like P(g_7NC$_6$N) and P(g_7NC$_8$N), trap PCAT-K out of the aqueous solution. For all P(g_7NC$_n$N):PCAT-K blends, the concentrates was 0.1 mg/ml.

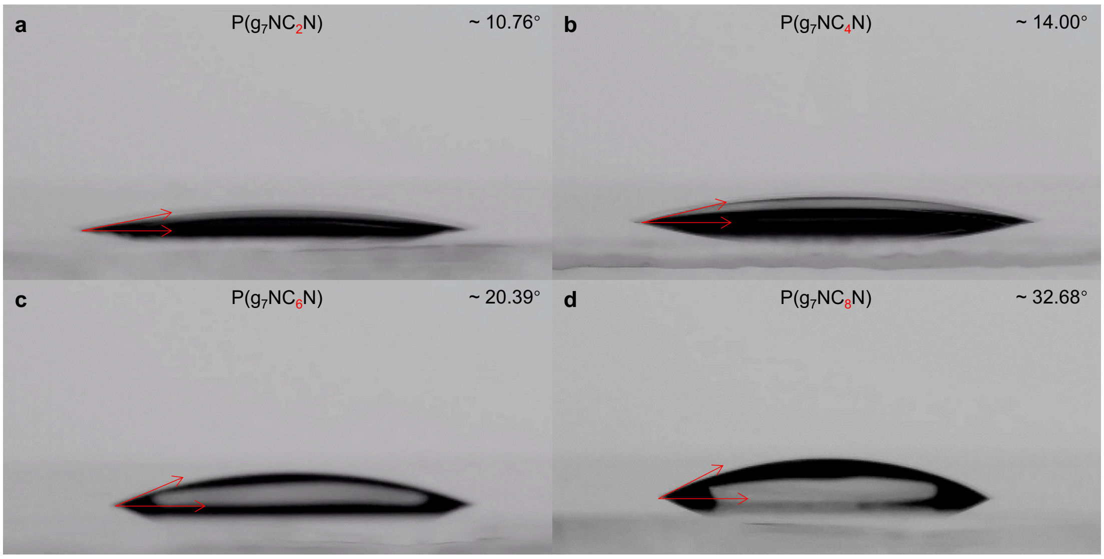

Supplementary Figure 25. Water contact angle on P(g_7NC$_n$N) polymer films with increasing alkyl chain length: (**a**) P(g_7NC$_2$N), (**b**) P(g_7NC$_4$N), (**c**) P(g_7NC$_6$N), and (**d**) P(g_7NC$_8$N).

.

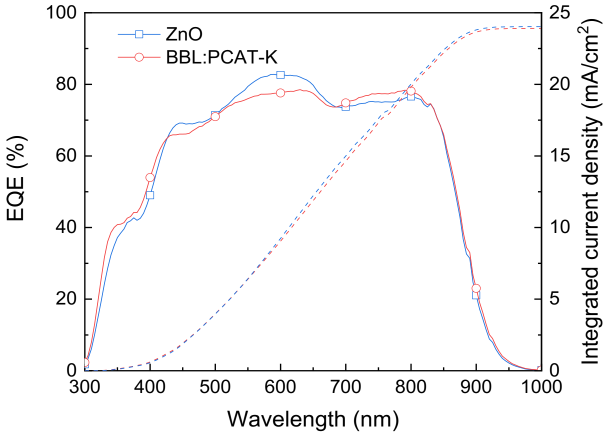

Supplementary Figure 26. EQE spectra of the inverted ITO/ETL/PM6:Y6:$PC_{71}BM$/MoO_3/Ag, where ETL is BBL:PCAT-K or ZnO as reference. The integrated current density is 23.91 and 24.03 mA cm^{-2} for BBL:PCAT-K and ZnO-based devices, respectively. These values are close to those obtained from the *J*-*V* curves.

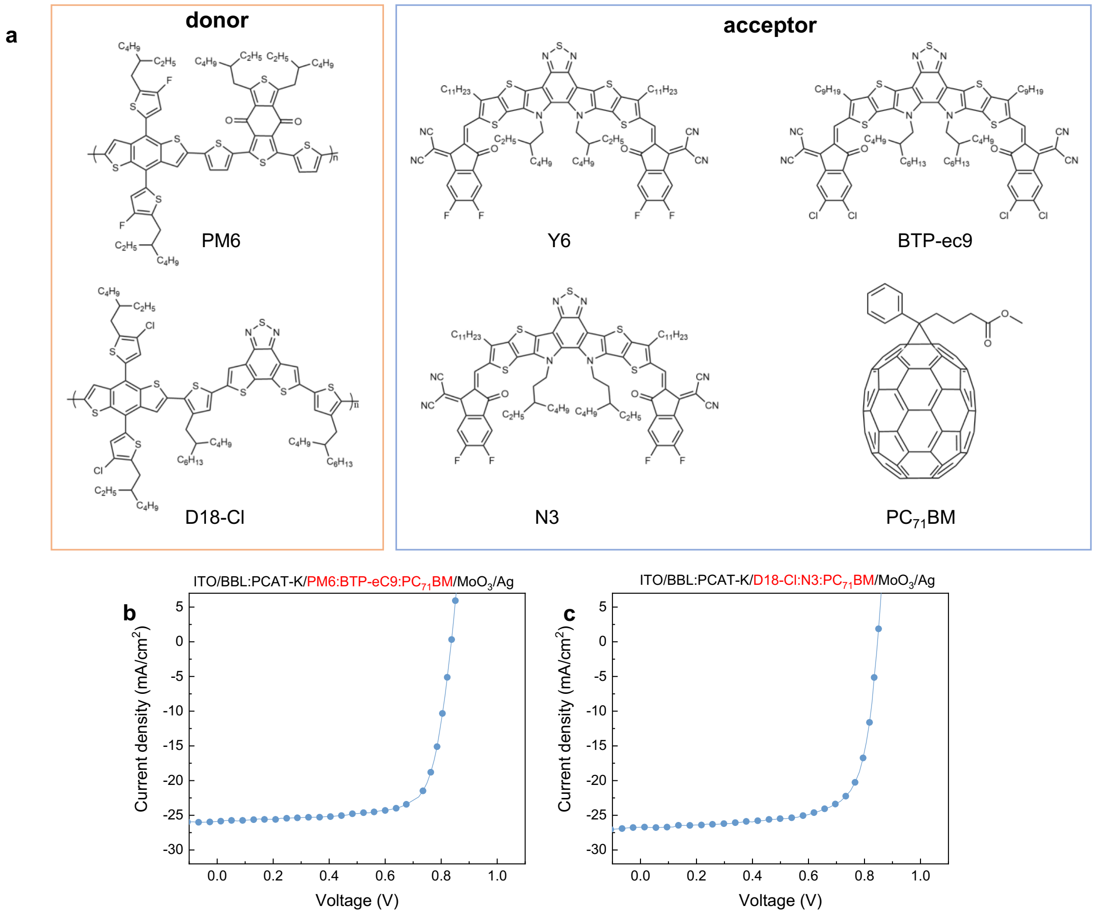

Supplementary Figure 27. (**a**) Chemical structure of the organic active materials used in this work. The *J*-*V* curves of the OSCs with structure ITO/BBL:PCAT-K/active layer/MoO_3/Ag, where the active layer is (**b**) PM6:BTP-ec9:$PC_{71}BM$ or (**c**) D18-Cl:N3:$PC_{71}BM$.

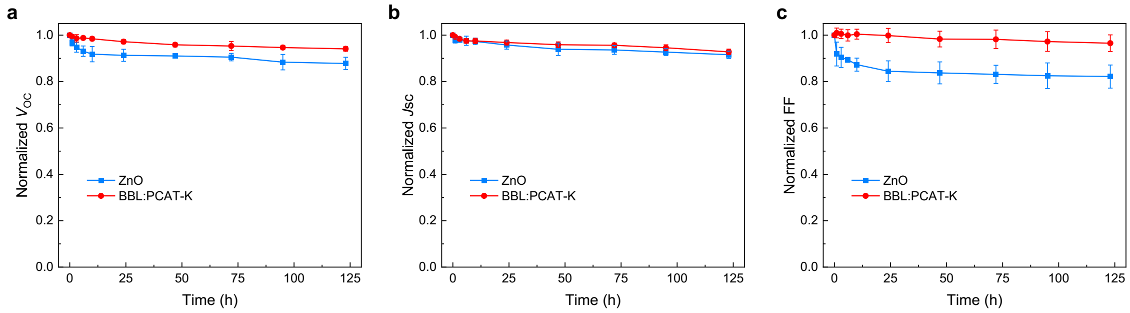

Supplementary Figure 28. Normalized (**a**) V_{OC}, (**b**) J_{SC}, and (**c**) FF of OSCs with the inverted structure ITO/ETL/PM6:Y6:$PC_{71}BM$/MoO_3/Ag, where ETL is either BBL:PCAT-K or ZnO, as a function of the illumination time under 100 mW cm^{-2} in an N_2-filled glovebox. Error bars indicate the SD of eight experimental replicates.

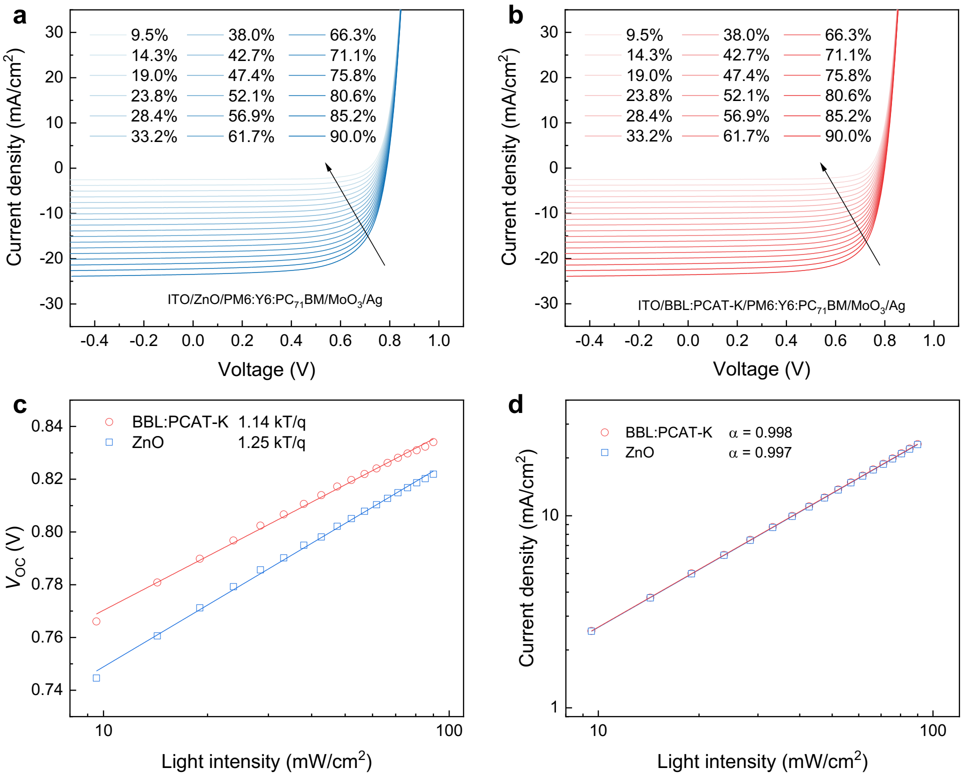

Supplementary Figure 29. *J-V* curves of OSCs with the inverted structure of ITO/ETL/PM6:Y6:$PC_{71}BM/MoO_3$/Ag, where ETL is (**a**) ZnO as reference or (**b**) BBL:PCAT-K. The relationship of (**c**) V_{OC} or (**d**) J_{SC} with light intensity extracted from *J-V* curves. The exponent α of the equation $J_{SC} \propto P^{\alpha}$ is close to 1 (0.998 for BBL:PCAT-K as ETL and 0.997 for ZnO as ETL), indicating negligible bimolecular recombination and that all carriers can be collected by the electrodes[10]. However, the slope of V_{OC} versus ln(*P*) in BBL:PCAT-K-based devices is smaller than that of ZnO-based reference device (1.14 *k*T/*q* vs 1.25 *k*T/*q*, where *k* is the Boltzmann constant, T is the absolute temperature and *q* is the elementary charge), suggesting suppression of trap-assisted recombination[11].

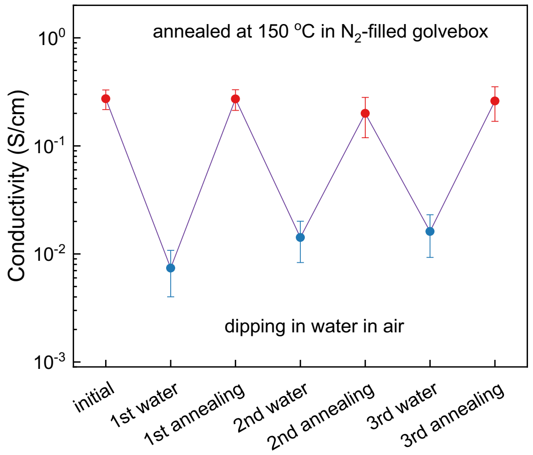

Supplementary Figure 30. Conductivity of BBL:PCAT-K (1:1) film dipped in water for 1 min and annealed in an N_2-filled glovebox. Error bars indicate the SD of ten samples. The reversible conductivity upon water/annealing treatment indicates that the decrease in conductivity is due to trapping/de-trapping of charges by water or oxygen[12].

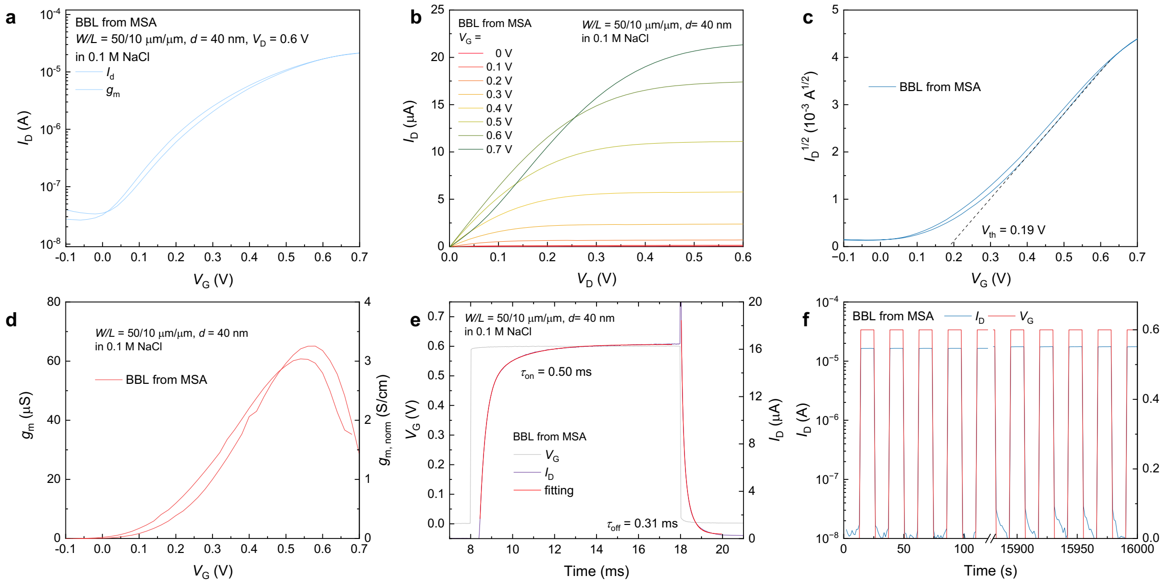

Supplementary Figure 31. Performance of pristine BBL-based OECTs processed from MSA. (**a**) Transfer curves, (**b**) output curves, (**c**) threshold voltage, (**d**) normalized transconductance, (**e**) transient response, and (**f**) long-term stability tests. The OECT's channel geometry is W = 50 μm, L = 10 μm, and d = 40 nm.

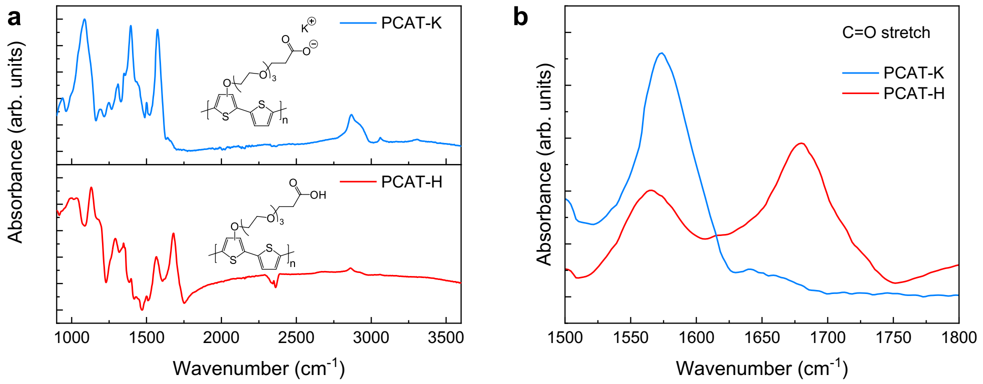

Supplementary Figure 32. (**a**) FT-IR spectra of PCAT-K (blue) and relative acid-treated form PCAT-H (red). Treatment: 10 mg/ml citric acid in IPA. (**b**) Zoom-in of the C=O stretching region at 1500-1800 cm^{-1}. The acid-treated PCAT-H is insoluble in water[13,14].

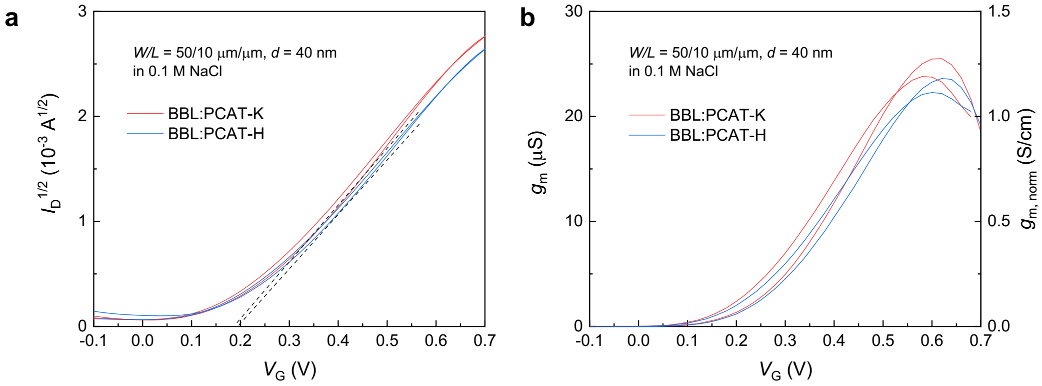

Supplementary Figure 33. (**a**) Threshold voltages and (**b**) normalized transconductance of BBL:PCAT-K and crosslinked BBL:PCAT-H based OECTs. The OECT's channel geometry is $W = 50$ μm, $L = 10$ μm, and $d = 40$ nm.

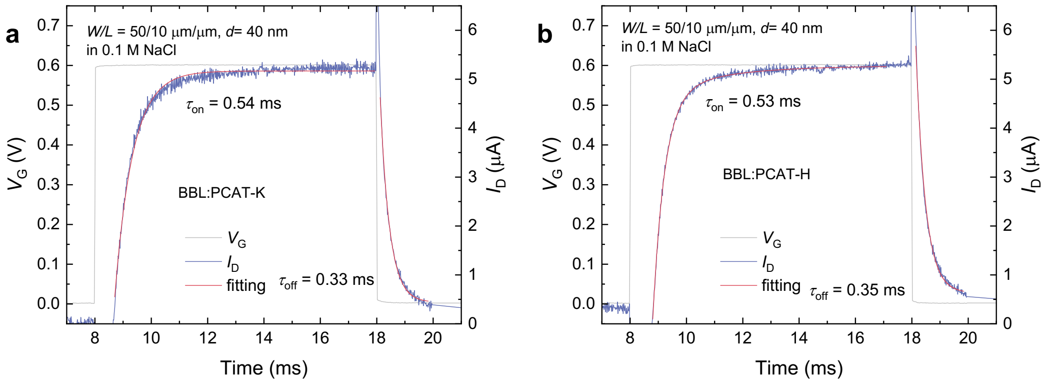

Supplementary Figure 34. Transient response of (**a**) BBL:PCAT-K and (**b**) crosslinked BBL:PCAT-H based OECTs. The OECT's channel geometry is $W = 50$ μm, $L = 10$ μm, and $d = 40$ nm.

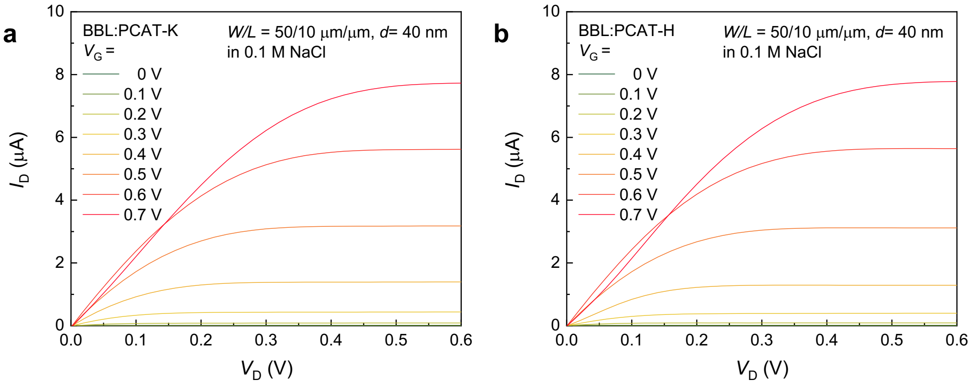

Supplementary Figure 35. Output curves of (**a**) BBL:PCAT-K and (**b**) crosslinked BBL:PCAT-H based OECTs. The OECT's channel geometry is $W = 50$ μm, $L = 10$ μm, and $d = 40$ nm.

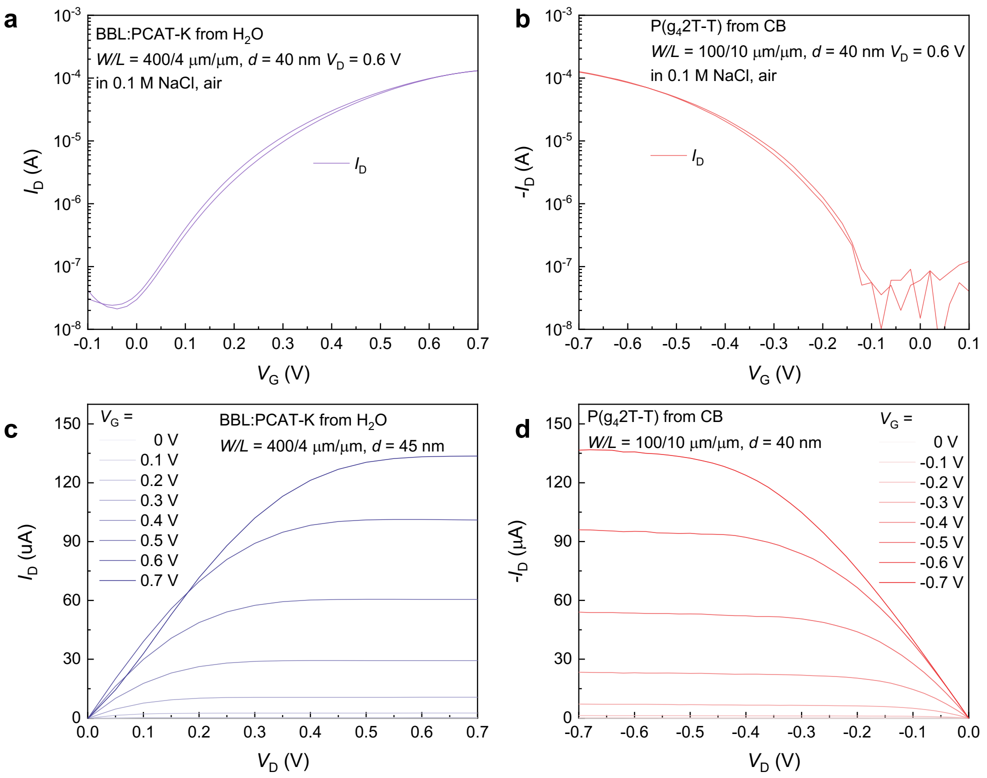

Supplementary Figure 36. Transfer curves of n-type BBL:PCAT-K (**a**) and p-type P(g42T-T) (**b**) based OECTs and output curves of (**c**) n-type BBL:PCAT-K and (**d**) p-type P($g_4$2T-T) based OECTs. The OECT's channel geometry is W/L = 400/4 µm/µm for the n-type OECTs and W/L = 100/10 µm/µm for the p-type OECTs, while the thickness of both BBL:PCAT-K and P($g_4$2T-T) films is about 40 nm.

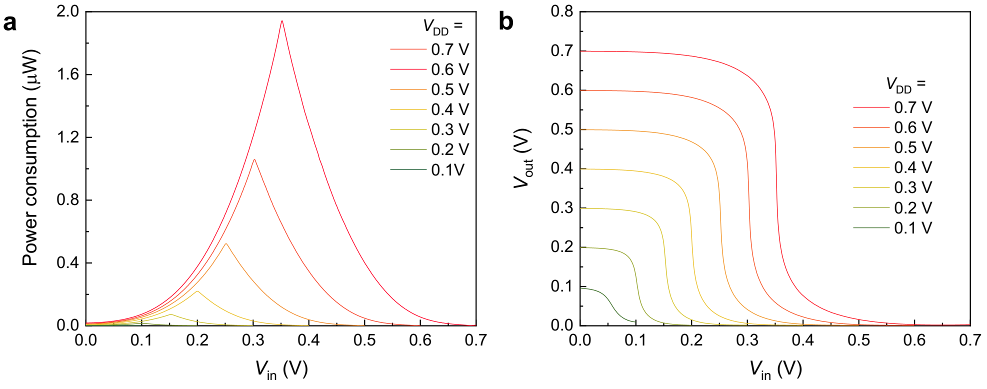

Supplementary Figure 37. (**a**) Power consumption and (**b**) voltage output of the complementary inverter at various supply voltages.

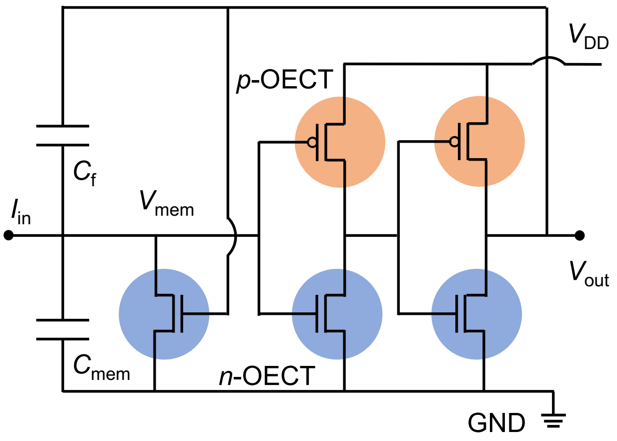

Supplementary Figure 38. Circuit of the leaky integrate and fire type organic electrochemical neuron.

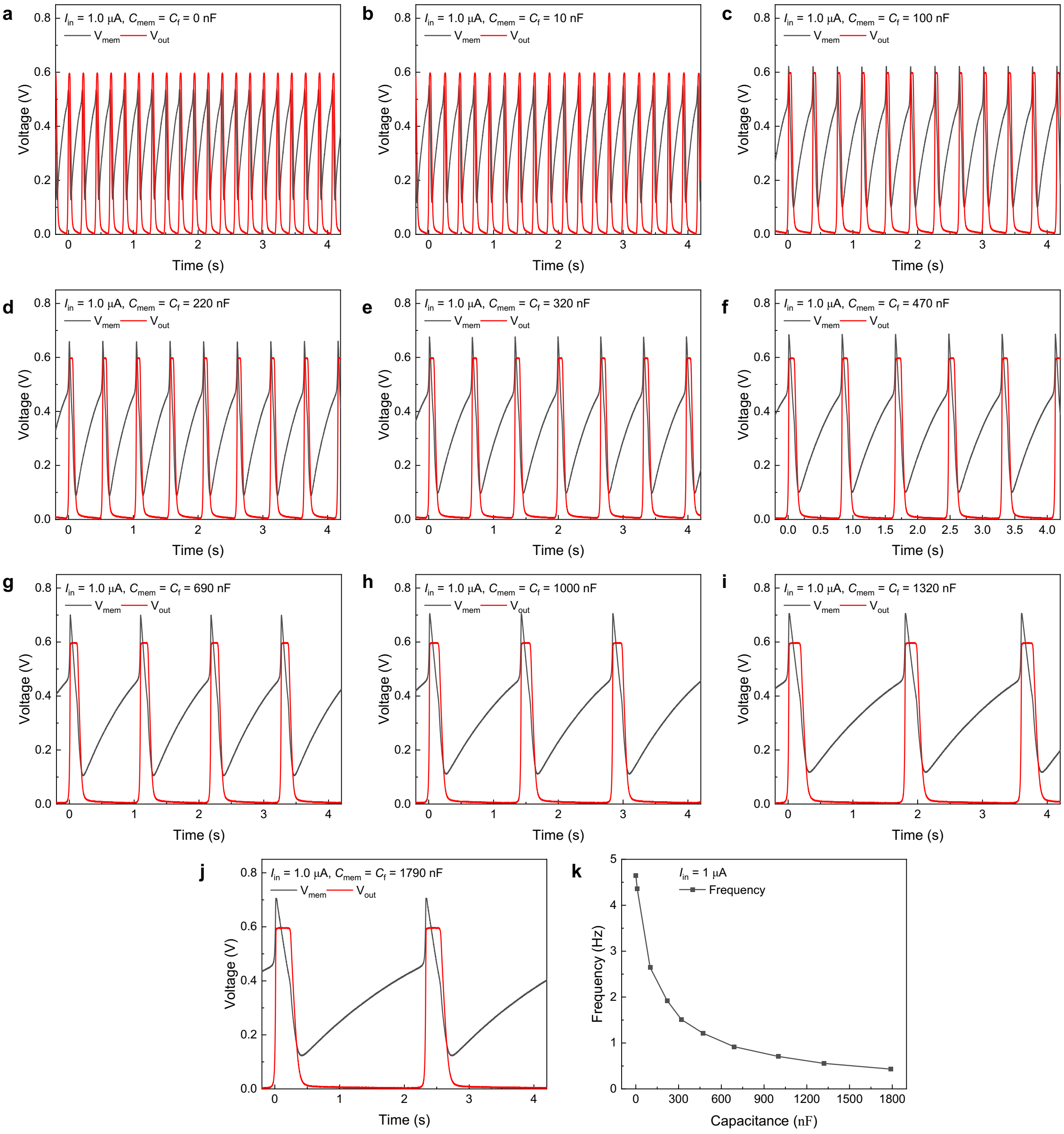

Supplementary Figure 39. Neuron characteristics recorded at a constant input current of 1 µA and increasing capacitance values: (**a**) C_{mem} = C_f = 0 nF, (**b**) C_{mem} = C_f = 10 nF, (**c**) C_{mem} = C_f = 100 nF, (**d**) C_{mem} = C_f = 220 nF, (**e**) C_{mem} = C_f = 320 nF, (**f**) C_{mem} = C_f = 470 nF, (**g**) C_{mem} = C_f = 690 nF, (**h**) C_{mem} = C_f = 1000 nF, (**i**) C_{mem} = C_f = 1320 nF, and (**j**) C_{mem} = C_f = 1790 nF. (**k**) Evolution of spiking frequency with capacitance.

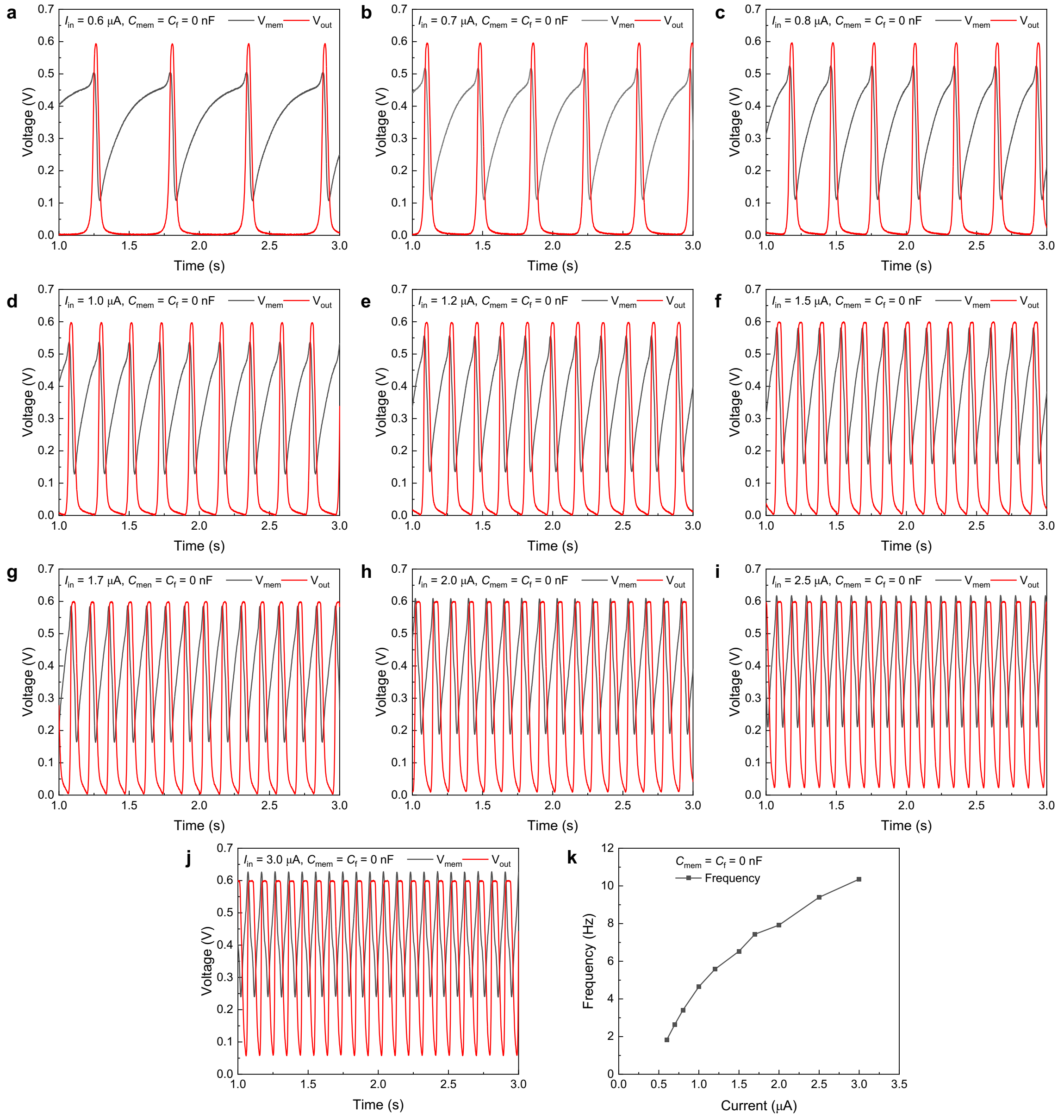

Supplement Figure 40. Neuron characteristics at a constant C_{mem} = C_{f} = 0 nF and increasing input current: (**a**) I_{in} = 0.6 μA, (**b**) I_{in} = 0.7 μA, (**c**) I_{in} = 0.8 μA, (**d**) I_{in} = 1.0 μA, (**e**) I_{in} = 1.2 μA, (**f**) I_{in} = 1.5 μA, (**g**) I_{in} = 1.7 μA, (**h**) I_{in} = 2.0 μA, (**i**) I_{in} = 2.5 μA, and (**j**) I_{in} = 3.0 μA. (**k**) Evolution of spiking frequency with current.

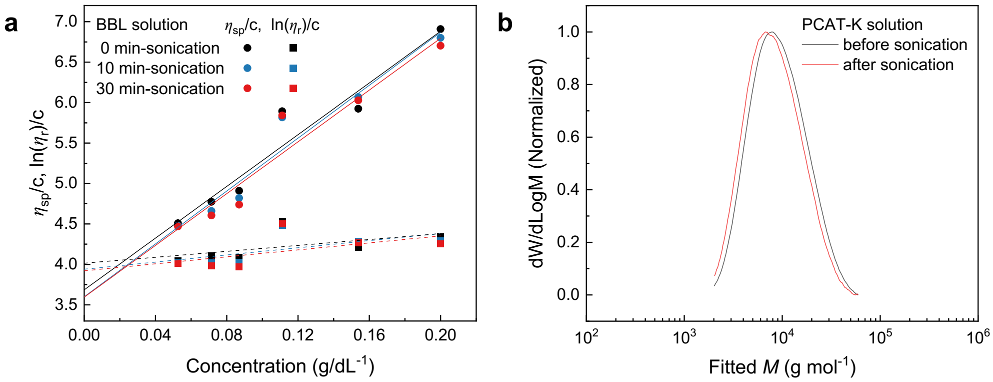

Supplementary Figure 41. Effect of continuous sonication (180 W, 30 min) on the molecular weight of BBL and PCAT-K. (**a**) The viscosity-average molecular weights (M_v) of BBL was estimated by measuring the intrinsic viscosity (η) of BBL solution (in MSA). The η and M_v follow the Mark-Houwink-Sakurada equation $\eta = KM_v^{\alpha}$, where K = 5.11 × 10^{-6} g dl^{-1} and α = 1.34. The viscosity was measured with an Ubbelohde type viscometer. The viscosity of BBL solution change from 3.85 to 3.76 dl g^{-1} after 30 min of sonication, resulting in a negligible variation (~2%) of the molecular weight. (**b**) The molecular weight of PCAT-K was measured by gel permeation chromatography (GPC). After 30 min sonication, the M_n of PCAT-K varies by ~4% (from 7.1 to 6.8 kDa).

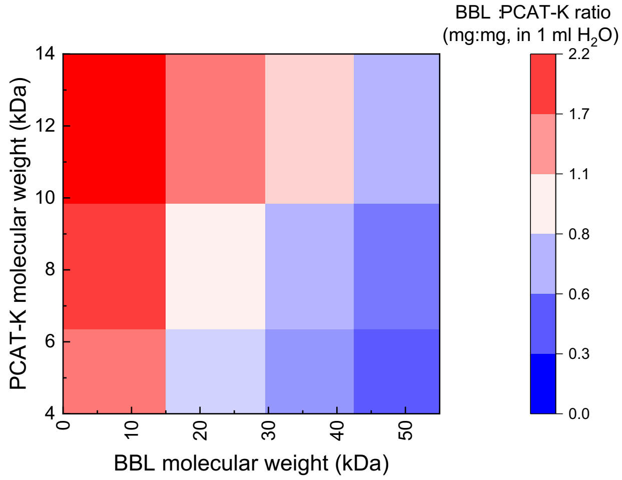

Supplementary Figure 42. BBL:PCAT-K ratio as a function of BBL and PCAT-K molecular weight.

Supplementary Table 1. Absolute spin counts and spin densities in the pristine BBL, PCAT-K, BBL:PCAT-K blend, and BBL/PCAT-K bilayer films as measured by EPR spectroscopy.

**Film**	**Abs. spin ($\times 10^{14}$ counts)**	**Volume ($\times 10^{-3}$ mm^{-3})**	**Spin density ($\times 10^{19}$ cm^{-3})**
BBL	0.44	39.57	0.11
PCAT-K	0.51	11.25	0.45
BBL/PCAT-K (bilayer)	2.52	N.A.	N.A.
BBL:PCAT-K (blend)	9.21	41.40	2.23

Supplementary Table 2. Photovoltaic parameters of the inverted OSCs with structure ITO/ETL/active layer/MoO_3/Ag measured under 100 mW cm^{-2} AM 1.5 G illumination. Average value and standard deviation data are calculated from 16 devices. The value in brackets is extracted from the device with the best efficiency.

Active layer	ETL	V_{OC} (V)	J_{SC} (mA cm^{-2})	FF	PCE (%)
PM6:Y6:$PC_{71}BM$	ZnO	0.82±0.01<br>(0.83)	25.36±0.41<br>(25.85)	0.70±0.02<br>(0.72)	14.67±0.56<br>(15.44)
	BBL:PCAT-K	0.83±0.01<br>(0.84)	25.27±0.45<br>(25.78)	0.71±0.02<br>(0.74)	15.38±0.61<br>(16.03)
PM6:BTP-ec9:$PC_{71}BM$	BBL:PCAT-K	0.83±0.01<br>(0.84)	25.73±0.27<br>(25.92)	0.71±0.02<br>(0.74)	15.48±0.48<br>(16.11)
D18-Cl:N3:$PC_{71}BM$	BBL:PCAT-K	0.84±0.01<br>(0.85)	26.58±0.25<br>(26.72)	0.71±0.02<br>(0.73)	15.86±0.60<br>(16.58)

Supplementary Table 3. List of water/alcohol-soluble conjugated polymers used as electron transport layers in OSCs.

Materials	Solvent	Work function (eV)	Conductivity (S/cm)	Carrier type	Ref.
BBL:PCAT-K	water	3.93	0.35	n	This work
PFN-Br	water/alcohol	3.82	~ 6×10^{-8}	n	15
PFN	methanol*	3.69	~ 1×10^{-6}	n	16
PBTA-FN	methanol*	3.55	~ 2×10^{-6}	n	16
PNDIT-F3N	methanol*	4.20	~ 7×10^{-6}	n	17
PNDIT-F3N-Br	water/alcohol	3.93	~ 2×10^{-5}	n	17
PNDIT-F3N–F	water/alcohol	4.08	N.A.	n	18
PNDIT-F3N–Cl	water/alcohol	4.08	N.A.	n	18
P(NDIDEG-T)	water/alcohol	4.29	1.75×10^{-6}	n	19
P(NDITEG-T)	water/alcohol	4.28	1.28×10^{-6}	n	19
P2G	water/alcohol	3.94	2.3×10^{-6}	n	20
PFP-F	water/alcohol	3.94	N.A.	n	21
PFP-Cl	water/alcohol	3.96	N.A.	n	21
PFP-Br	water/alcohol	3.66	N.A.	n	21
PFP-I	water/alcohol	3.74	N.A.	n	21
PDPPTPT-N^+Br^-	water/alcohol	4.52	8.87×10^{-4}	n	22
PDPPTPf$_2$T-N^+Br^-	water/alcohol	4.31	5.31×10^{-4}	n	22
PDPPTPf$_4$T-N^+Br^-	water/alcohol	4.18	2.58×10^{-4}	n	22
WPF-oxy-F	water/alcohol	4.12	N.A.	n	23
WPF-6-oxy-F	water/alcohol	3.99	N.A.	n	23
PEDOT:PSS-TBA	water	4.11	2.39	p	24

*with acetic acid as the additive.

Supplementary Table 4. Performance of OECTwith different BBL formulations.

Formulation	Solvent	V_{th} (V)	$g_{m,norm}$ (S/cm)	τ_{ON} (ms)	τ_{OFF} (ms)	I_{ON}/I_{OFF}	Carrier type	Ref.
BBL:PCAT-K[a]	Water	0.19	1.28	0.54	0.33	1.9×10^3	n	This work
BBL:PCAT-H[a]	Water	0.20	1.18	0.53	0.35	1.6×10^3	n	This work
BBL[a]	MSA	0.19	3.53	0.50	0.31	2.1×10^3	n	This work
BBL*[,a]	MSA	0.21	0.359	900	200	6.0×10^3	n	25
BBL_{15}[a]	MSA	0.27	0.617	0.89	0.79	2.9×10^3	n	26
BBL_{60}[a]	MSA	0.21	1.92	0.52	0.27	8.3×10^4	n	26
BBL_{98}[a]	MSA	0.18	4.04	0.43	0.23	2.0×10^5	n	26
BBL_{152}[a]	MSA	0.15	11.1	0.38	0.16	4.4×10^5	n	26
BBL:MWCNT 100:1[a]	IPA	N.A.	0.234	37	27	~ 10^3	n	27
BBL:MWCNT 10:1[a]	IPA	N.A.	0.335	68	14	~ 10^3	n	27
BBL:MWCNT 1:1[a]	IPA	N.A.	0.182	34	27	~ 10^2	n	27
BBL+MWCNT 100+1[a]	IPA	N.A.	0.117	N.A.	N.A.	~ 10^3	n	27
BBL+MWCNT 10+1[a]	IPA	N.A.	0.086	N.A.	N.A.	~ 10^2	n	27
BBL+MWCNT 1+1[a]	IPA	N.A.	0.068	N.A.	N.A.	~ 10^2	n	27
BBL:PEI[b]	Ethanol	N.A.	2.28	167	11	~ 10^3	n	28
PTTBL:BBL 3:1[a]	MSA	0.26^n<br>0.43^p	0.43^n<br>0.41^p	1.72^n<br>3.05^p	0.38^n<br>1.95^p	~ $(10^3)^n$<br>~ $(10^3)^p$	p/n	29
PTTBL:BBL 2:1[a]	MSA	N.A.	0.50^n<br>0.28^p	N.A.	N.A.	~ $(10^3)^n$<br>~ $(10^3)^p$	p/n	29
PTTBL:BBL 1:1[a]	MSA	N.A.	0.62^n<br>0.11^p	N.A.	N.A.	~ $(10^3)^n$<br>~ $(10^3)^p$	p/n	29

[a]Accumulation and [b]depletion mode of operation. *First reported BBL-based OECT. IPA: isopropanol. MSA: methanesulfonic acid. BBL:MWCNT refers as "co-ink". BBL+MWCNT refers to "mix-ink". The subscripted number refer to the repeating unit. The superscript n and p refer to the characteristics of the devices working on the n- or p- mode.

Supplementary Table 5. Performance of OECT-based complementary inverters.

V_{DD} (V)	V_M (V)	Gain (V/V)	V_{IL} (V)	V_{IH} (V)	NM_L (V)	NM_H (V)	NM (%)	P_{static} (nW)	$P_{dynamic}$ (μW)
0.7	0.353	54.8	0.298	0.412	0.298	0.288	83.7	18.3	1.94
0.6	0.303	42.7	0.257	0.356	0.257	0.244	83.5	12.4	1.06
0.5	0.252	33.2	0.214	0.300	0.214	0.200	82.8	9.39	0.53
0.4	0.201	22.7	0.169	0.241	0.169	0.159	82.0	6.92	0.22
0.3	0.154	17.0	0.128	0.184	0.124	0.116	80.0	4.16	0.071
0.2	0.103	9.1	0.082	0.125	0.082	0.075	78.5	1.56	0.0159
0.1	0.055	2.3	0.040	0.076	0.040	0.024	64.0	0.32	0.00041

Here V_{IH} and V_{IL} are the input voltages HIGH and LOW at the operation points of $\frac{\partial V_{out}}{\partial V_{in}} = 1$ at the voltage transfer characteristics of the inverter, high and low noise margins (NM_H and NM_L) defined as $NM_H = V_{DD} - V_{IH}$, $NM_L = V_{IL}$, and total $NM = NM_H + NM_L$. The NM is expressed as a percentage of V_{DD}.

Supplementary References

1. Duan, H. *et al.* Facile synthesis of water-dispersible poly(3-hexylthiophene) nanoparticles with high yield and excellent colloidal stability. *iScience* **25**, 104220 (2022).

2. Fraleoni-Morgera, A., Marazzita, S., Frascaro, D. & Setti, L. Influence of a non-ionic surfactant on the UV–vis absorption features of regioregular head-to-tail poly(3-hexylthiophene) in water-based dispersions. *Synth. Met.* **147**, 149–154 (2004).

3. Tong, J., Han, C., Hao, X., Qin, X. & Li, B. Conductive Polyacrylic Acid-Polyaniline as a Multifunctional Binder for Stable Organic Quinone Electrodes of Lithium-Ion Batteries. *ACS Appl. Mater. Interfaces* **12**, 39630–39638 (2020).

4. Hofmann, A. I. *et al.* How To Choose Polyelectrolytes for Aqueous Dispersions of Conducting PEDOT Complexes. *Macromolecules* **50**, 1959–1969 (2017).

5. Carli, S. *et al.* Water-Based PEDOT:Nafion Dispersion for Organic Bioelectronics. *ACS Appl. Mater. Interfaces* **12**, 29807–29817 (2020).

6. Lei, J. *et al.* Single-dispersed polyoxometalate clusters embedded on multilayer graphene as a bifunctional electrocatalyst for efficient Li-S batteries. *Nat. Commun.* **13**, 202 (2022).

7. Yohannes, T. *et al.* Multiple electrochemical doping-induced insulator-to-conductor transitions observed in the conjugated ladder polymer polybenzimidazobenzophenanthroline (BBL). *J. Phys. Chem. B* **104**, 9430–9437 (2000).

8. Fazzi, D., Fabiano, S., Ruoko, T. P., Meerholz, K. & Negri, F. Polarons in π-conjugated ladder-type polymers: A broken symmetry density functional description. *J. Mater. Chem. C* **7**, 12876–12885 (2019).

9. Yin, J., Wang, Z., Fazzi, D., Shen, Z. & Soci, C. First-Principles Study of the Nuclear Dynamics of Doped Conjugated Polymers. *J. Phys. Chem. C* **120**, 1994–2001 (2016).

10. Cowan, S. R., Roy, A. & Heeger, A. J. Recombination in polymer-fullerene bulk heterojunction solar cells. *Phys. Rev. B - Condens. Matter Mater. Phys.* **82**, 245207 (2010).

11. Yao, J. *et al.* Cathode engineering with perylene-diimide interlayer enabling over 17% efficiency single-junction organic solar cells. *Nat. Commun.* **11**, 1–10 (2020).

12. Nikolka, M. *et al.* High operational and environmental stability of high-mobility conjugated polymer field-effect transistors through the use of molecular additives. *Nat. Mater.* **16**, 356–362 (2017).

13. Ponder, J. F. J., Österholm, A. M. & Reynolds, J. R. Conjugated Polyelectrolytes as Water Processable Precursors to Aqueous Compatible Redox Active Polymers for Diverse Applications: Electrochromism, Charge Storage, and Biocompatible Organic Electronics. *Chem. Mater.* **29**, 4385–4392 (2017).

14. Shi, P., Amb, C. M., Dyer, A. L. & Reynolds, J. R. Fast Switching Water Processable Electrochromic Polymers. *ACS Appl. Mater. Interfaces* **4**, 6512–6521 (2012).

15. Tang, H. *et al.* Organic diradicals enabled N-type self-doped conjugated polyelectrolyte with high transparency and enhanced conductivity. *Giant* **6**, 100053 (2021).

16. Wang, S. *et al.* Self-doping small molecular conjugated electrolytes enabled by n-type side chains for highly efficient non-fullerene polymer solar cells. *J. Mater. Chem. A* **6**, 22503–22507 (2018).

17. Wu, Z. *et al.* n-Type Water/Alcohol-Soluble Naphthalene Diimide-Based Conjugated Polymers for High-Performance Polymer Solar Cells. *J. Am. Chem. Soc.* **138**, 2004–2013 (2016).

18. Chen, Z. *et al.* Counterion-tunable n-type conjugated polyelectrolytes for the interface engineering of efficient polymer solar cells. *J. Mater. Chem. A* **5**, 19447–19455 (2017).

19. Lee, S. *et al.* Electron Transport Layers Based on Oligo(ethylene glycol)-Incorporated Polymers Enabling Reproducible Fabrication of High-Performance Organic Solar Cells. *Macromolecules* **54**, 7102–7112 (2021).

20. Sharma, A. *et al.* A Nonionic Alcohol Soluble Polymer Cathode Interlayer Enables Efficient Organic and Perovskite Solar Cells. *Chem. Mater.* **33**, 8602–8611 (2021).

21. Hu, Z. *et al.* Phosphonium conjugated polyelectrolytes as interface materials for efficient polymer solar cells. *Org. Electron.* **57**, 151–157 (2018).

22. Jin, X. *et al.* Fluorine-induced self-doping and spatial conformation in alcohol-soluble interlayers for highly-efficient polymer solar cells. *J. Mater. Chem. A* **6**, 423–433 (2018).

23. Oh, S.-H. *et al.* Water-Soluble Polyfluorenes as an Interfacial Layer Leading to Cathode-Independent High Performance of Organic Solar Cells. *Adv. Funct. Mater.* **20**, 1977–1983 (2010).

24. Liu, T. *et al.* Low-Work-Function PEDOT Formula as a Stable Interlayer and Cathode for Organic Solar Cells. *Adv. Funct. Mater.* **31**, 2107250 (2021).

25. Sun, H. *et al.* Complementary Logic Circuits Based on High-Performance n-Type Organic Electrochemical Transistors. *Adv. Mater.* **30**, 1704916 (2018).

26. Wu, H. Y. *et al.* Influence of Molecular Weight on the Organic Electrochemical Transistor Performance of Ladder-Type Conjugated Polymers. *Adv. Mater.* **34**, 2106235 (2022).

27. Zhang, S. *et al.* Synergistic Effect of Multi-Walled Carbon Nanotubes and Ladder-Type Conjugated Polymers on the Performance of N-Type Organic Electrochemical Transistors. *Adv. Funct. Mater.* **32**, 2106447 (2022).

28. Yang, C. Y. *et al.* A high-conductivity n-type polymeric ink for printed electronics. *Nat. Commun.* **12**, 1–8 (2021).

29. Wu, X. *et al.* All-Polymer Bulk-Heterojunction Organic Electrochemical Transistors with Balanced Ionic and Electronic Transport. *Adv. Mater.* **34**, 2206118 (2022).
